# Supplementary material for: A transposon insertion in the 5′ UTR of OsPT1 reprograms its expression pattern and promotes cadmium accumulation in rice grains
Source: Plant Commun. 2025 Oct 15;7(1):101566. doi: 10.1016/j.xplc.2025.101566 (PMC12902297; doi:10.1016/j.xplc.2025.101566)
Supplement: Document S1. Supplemental Figures 1–10 [file mmc1.pdf]

Plant Communications, Volume *7*

Supplemental information

A transposon insertion in the 5′ UTR of *OsPT1* reprograms its expression pattern and promotes cadmium accumulation in rice grains

Shasha Peng, Dan Wang, Jinling Liu, Su Jiang, Yuchen Xu, Yufei Deng, Xiaolong Zhou, Fangzhi Hu, Zhuo Liu, Ye Peng, Hejun Ao, Yinghui Xiao, Jiurong Wang, Junliang Zhao, Bin Liu, Keke Yi, Lianyang Bai, Guo-Liang Wang, and Houxiang Kang

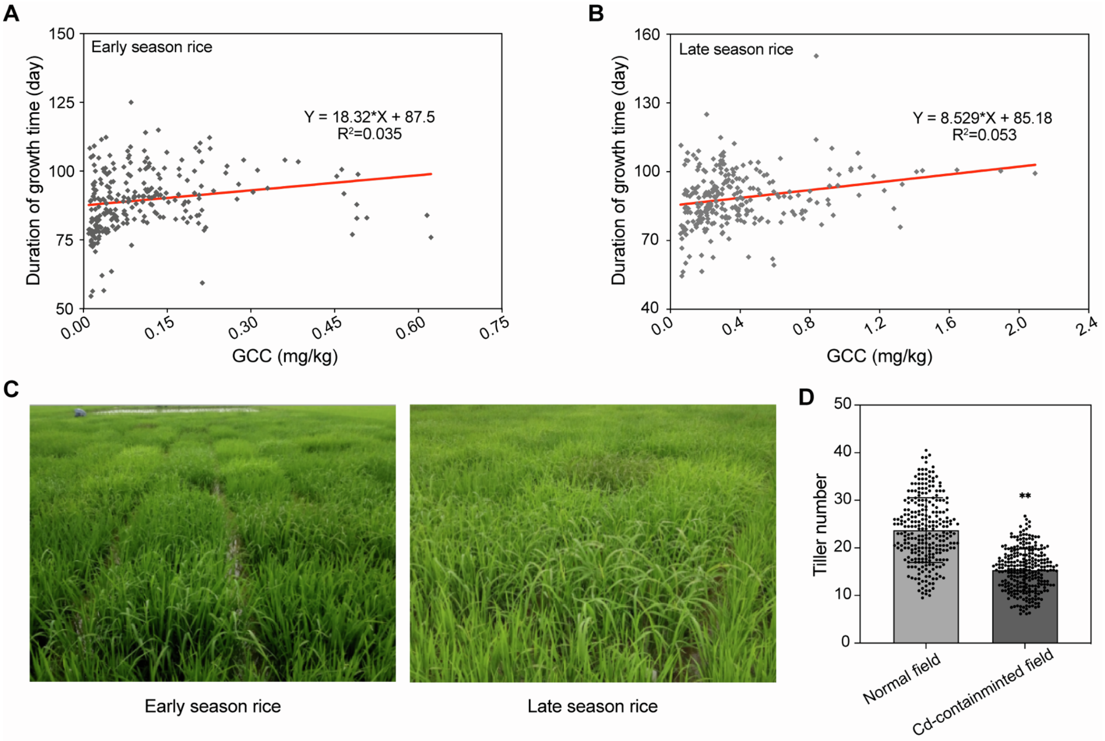

Figure S1. The correlation analysis between GCC and the growth period. (A and B) Scatter plots showing the relationship between GCC and growth duration for early-season rice **(A)** and late-season rice **(B)**. The fitted linear regression lines and corresponding equations are shown.

(**C**) Field photographs of RDP1 rice cultivars after transplanting: early-season rice (left), late-season rice (right).

(**D**) Tiller number of RDP1 cultivars grown in normal (left) and Cd-contaminated (right) fields. Each dot represents a single accession. Error bars represent SD; the asterisks denote significant differences according to Student's *t*-test (**P <0.01).

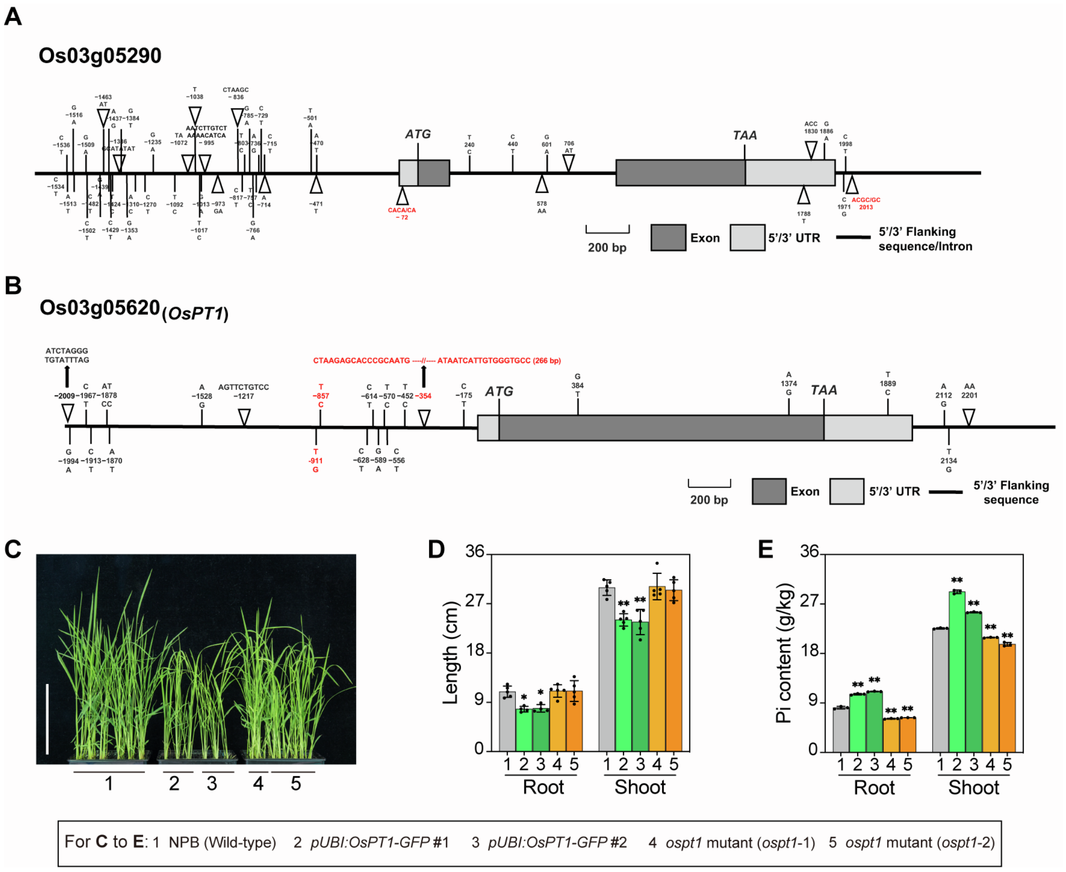

Figure S2. The polymorphisms were detected in two transporter genes in LAGCC4.

(A and B) Gene structure diagrams showing the polymorphisms identified by PCR and Sanger sequencing in Os03g05290 **(A)** and Os03g05620 (*OsPT1*) **(B)**. Black boxes indicate exons; gray boxes indicate 5′/3′ UTR; lines represent flanking sequences and intron.

(C) Representative phenotypes of 2-week-old wild-type, *ospt1* mutants, and *OsPT1* overexpression (*pUBI:OsPT1-GFP*) lines transferred to hydroponic growth conditions. Scale bar = 10 cm.

(D) *OsPT1* overexpression lines have shorter root and shoot lengths. Error bars represent SD; asterisks denote significant differences according to Student's *t*-test (*$P < 0.05$, **$P < 0.01$).

(E) *OsPT1* overexpression lines show elevated phosphate (Pi) content in roots and shoots. In contrast, *ospt1* mutants have reduced Pi content in both tissues. Error bars represent SD; asterisks denote significant differences according to Student's t-test (*$P < 0.05$, **$P < 0.01$).

A

Group	Accessions \ Position	ATG-1223	ATG-911	ATG-857	ATG-602	ATG-354	ATG+384	ATG+1374
High GCC accessions	*NSFTV_398*	8 bp +	T	T	C	266 bp +	G	A
	NSFTV_355	8 bp +	T	T	C	266 bp +	G	A
	NSFTV_353	8 bp +	T	T	C	266 bp +	G	A
	NSFTV_154	8 bp +	T	T	C	266 bp +	G	A
	NSFTV_156	8 bp +	T	T	C	266 bp +	G	A
	NSFTV_074	8 bp +	T	T	C	266 bp +	G	A
	NSFTV_072	8 bp +	T	T	C	266 bp +	G	A
	NSFTV_633	8 bp +	T	T	C	266 bp +	G	A
	NSFTV_076	8 bp +	T	T	C	266 bp +	G	A
	NSFTV_071	8 bp +	T	T	C	266 bp +	G	A
Low GCC accessions	*NSFTV_173*	-	G	C	C	-	T	G
	NSFTV_365	-	G	C	C	-	T	G
	NSFTV_064	-	G	C	C	-	T	G
	NSFTV_287	-	G	C	C	-	T	G
	NSFTV_177	-	G	C	C	-	T	G
	NSFTV_121	-	G	C	T	-	T	G
	NSFTV_629	-	G	C	C	-	T	G
	NSFTV_115	-	G	C	C	-	T	G
	NSFTV_224	-	G	C	C	-	T	G
	NSFTV_073	-	G	C	C	-	T	G
	NSFTV_033	-	G	C	C	-	T	G
	NSFTV_307	-	G	C	C	-	T	G
	NSFTV_265	-	G	C	C	-	T	G
	NSFTV_339	-	G	C	T	-	T	G
	NSFTV_091	-	G	C	C	-	T	G
	NSFTV_335	-	G	C	T	-	T	G

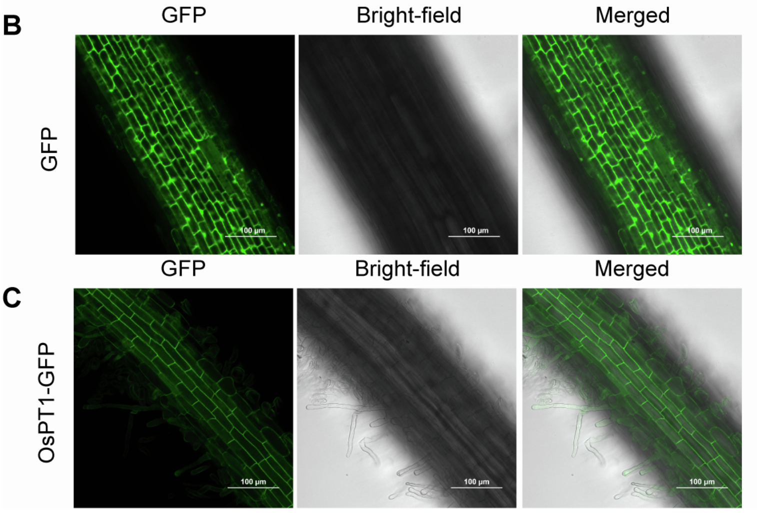

Figure S3. Haplotype variation and subcellular localization analysis of OsPT1.

(A) Haplotype analysis of *OsPT1* in 10 rice accessions with high GCC (in blue) and 16 rice accessions with low GCC (in green).

(B and C) Fluorescence microscopy showing subcellular localization of GFP **(B)** and OsPT1-GFP **(C)** in rice root tip cells.

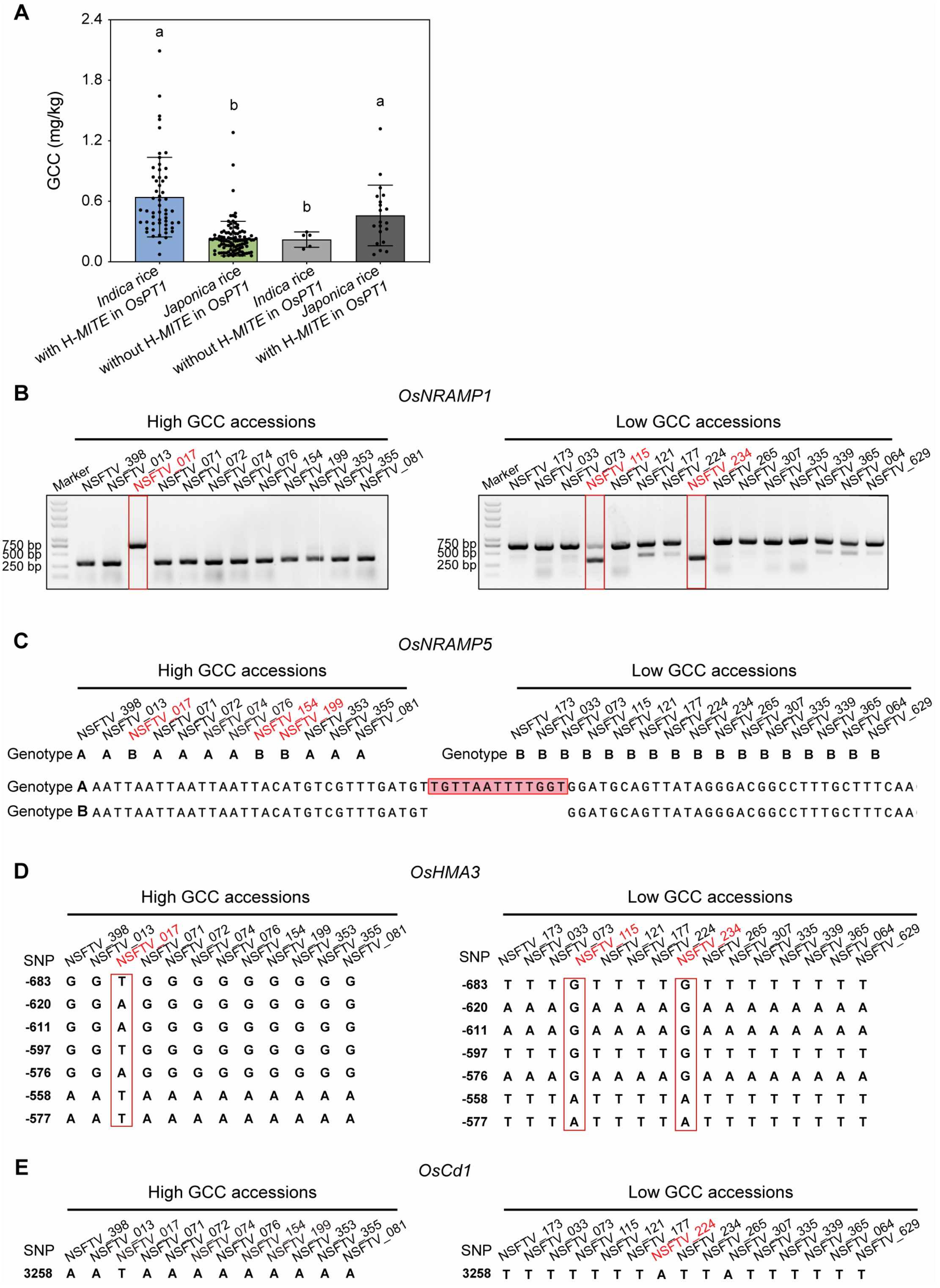

Figure S4. GCC of the RDP1 rice with or without H-*MITE* in *OsPT1* and genotype analysis of rice accessions for four key Cd-associated transporters.

(A) Histogram of average GCC for *Indica* rice with H-*MITE* in *OsPT1*, *Japonica* rice without H-*MITE* in *OsPT1, Indica* rice without H-*MITE* in *OsPT1,* and *Japonica* rice with H-*MITE* in *OsPT1*. Error bars represent SD; the "a" and "b" denote significant differences, as determined by one-way ANOVA with Tukey's HSD test.

(B) Genotypes of *OsNRAMP1* in representative accessions with high (left) and low (right) GCC, shown by gel electrophoresis.
(C) Genotypes of *OsNRAMP5* in high GCC accessions (left) and low GCC accessions (right). A and B represent the different genotypes, respectively.
(D) Genotypes of *OsHMA3* in high GCC accessions (left) and low GCC accessions (right).
(E) Genotypes of *OsCd1* in high GCC accessions (left) and low GCC accessions (right).

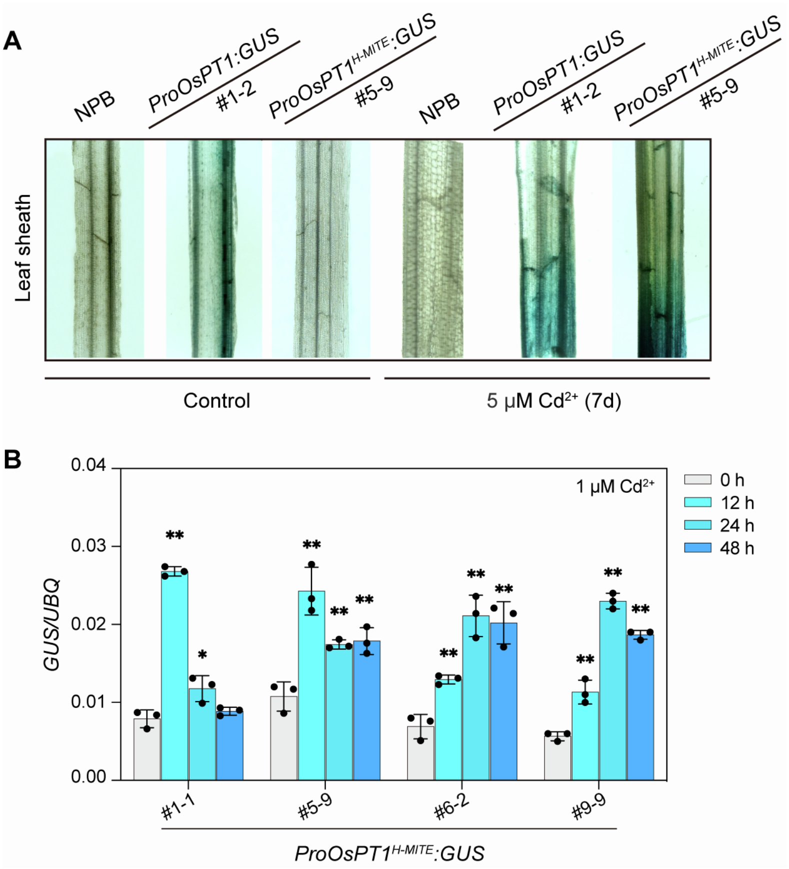

Figure S5. Cd treatment induces *GUS* transcription in stable transgenic rice plants of *ProOsPT1*$^{H\text{-}MITE}$:*GUS* but not *ProOsPT1*:*GUS*.

(A) GUS staining of leaf sheaths in wild-type (NPB), *ProOsPT1*:*GUS* and *ProOsPT1*$^{H\text{-}MITE}$:*GUS* transgenic seedlings; images on the left are from the control (no Cd treatment), images on the right are from seedlings exposed to Cd for 7 days.

(B) qRT-PCR analysis of GUS expression in four independent *ProOsPT1*$^{H\text{-}MITE}$ T_2 lines (#1-1, #5-9, #6-2 and #9-9) under 1 μM Cd^{2+} stress condition. Error bars indicate SD; asterisks denote significant differences compared with 0 h (*P < 0.05, **P < 0.01, *t*-test). Different shades represent different Cd exposure durations.

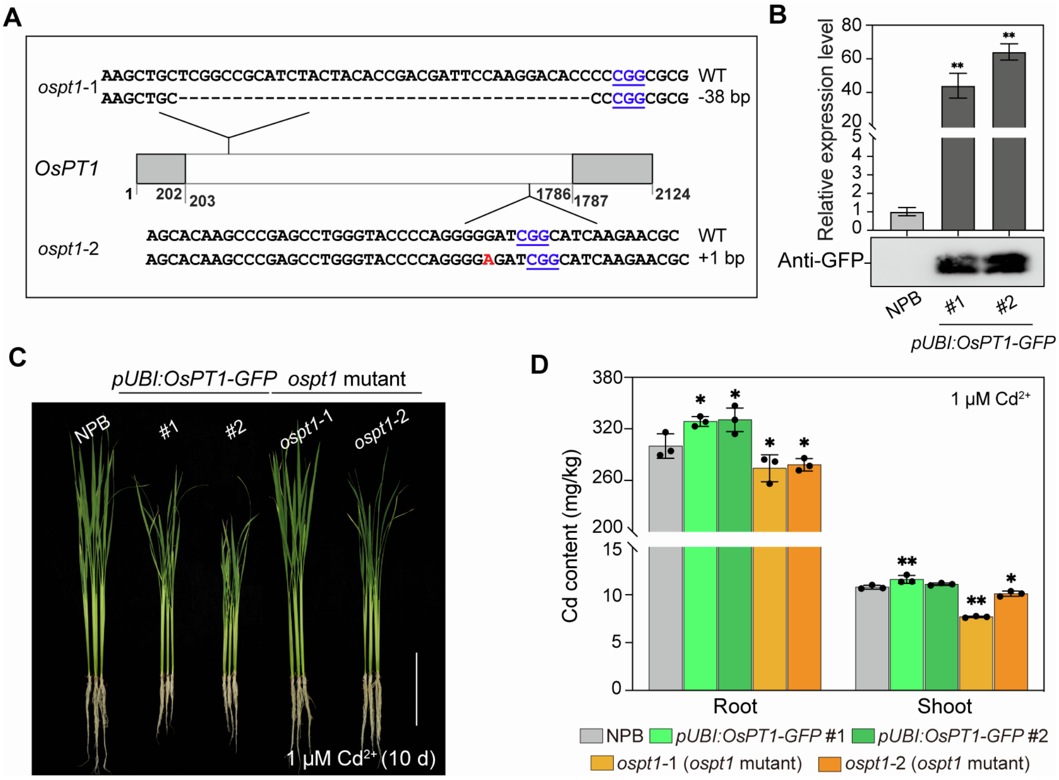

Figure S6. Overexpression of *OsPT1* causes high Cd accumulation in rice seedlings.

(A) Targeted mutagenesis of *OsPT1* using CRISPR-Cas9 mediated genome editing. Two independent gene editing sites were designed (sgRNA sequences are given in the figure). Two homozygous mutants (*ospt1-1* and *ospt1-2*) were obtained, harboring a 38-bp deletion (*ospt1-1*) or a 1-bp insertion (*ospt1-2*).

(B) *OsPT1* transcript levels and OsPT1 protein abundance in transgenic plants *OsPT1* overexpressing (*pUBI:OsPT1-GFP*#1 and *pUBI:OsPT1-GFP*#2). Protein abundance was determined by immunoblot with anti-GFP antibody.

(C and D) Phenotypes of wild type, *ospt1* mutant, and *OsPT1*-overexpressing (*pUBI:OsPT1-GFP*) lines, 2-week-old rice seedlings transferred to hydroponic growth conditions with 1 μM Cd^{2+} for 10 days **(C)**. Cd content in roots and shoots after treatment with 1 μM Cd^{2+} for 10 days in *OsPT1*-overexpressing and *ospt1* mutant plants **(D)**. Error bars represent SD; asterisks denote significant differences according to Student's *t*-test (**P* < 0.05, ***P* < 0.01).

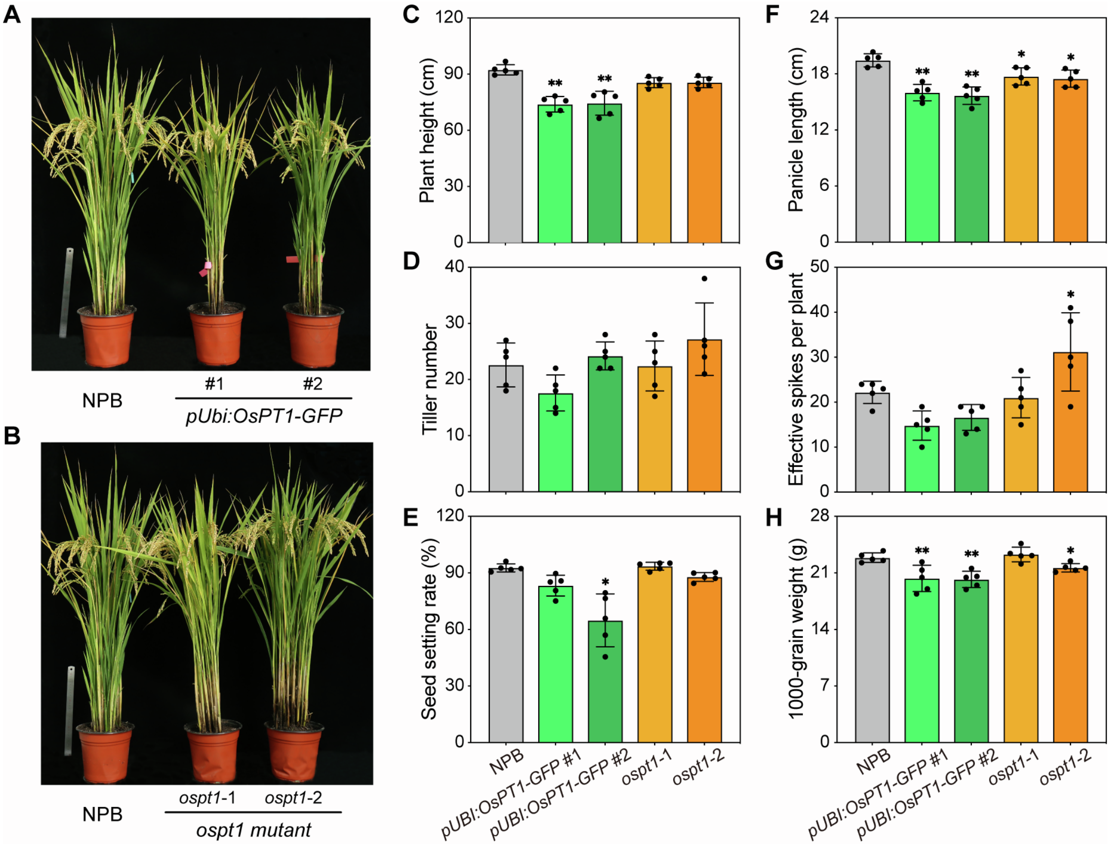

Figure S7. The agronomic traits of *OsPT1* overexpression and *ospt1* mutant plants.

(A and B) Phenotypes of wild type, *OsPT1*-overexpression **(A)**, and *ospt1* mutant **(B)** plants grown in the soil.

(C to H) The agronomic traits of wild type, *OsPT1*-overexpression, and *ospt1* mutant plants. Plant height **(C)**, tiller number **(D)**, seed setting **(E)**, panicle length **(F)**, effective spikes per plant **(G)**, and 1000-grain weight **(H)**. Error bars represent SD; asterisks denote significant differences according to Student's *t*-test (*$P < 0.05$, **$P < 0.01$).

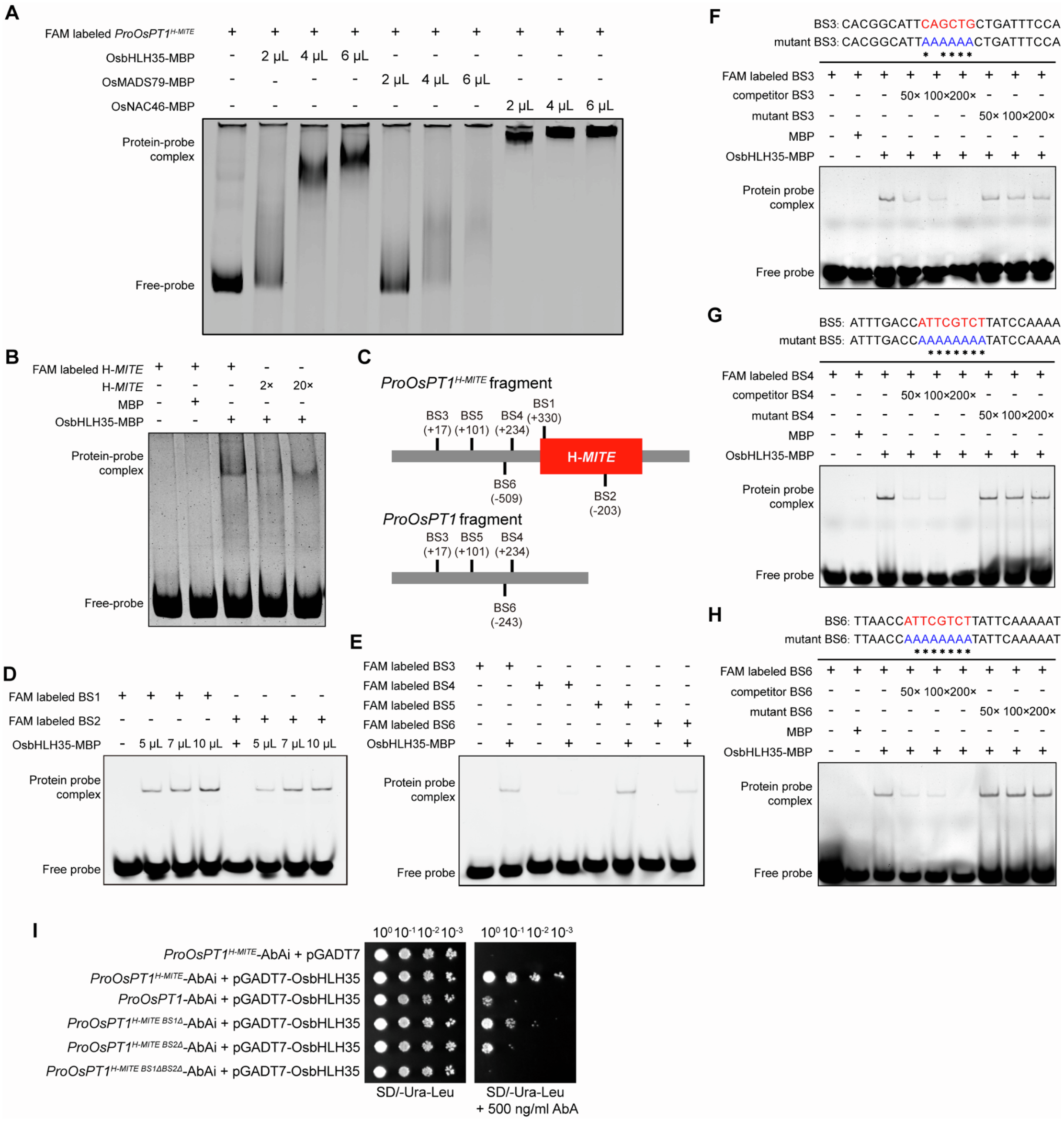

Figure S8. OsbHLH35 binds directly to *ProOsPT1$^{H\text{-}MITE}$*.

(A) EMSAs showing binding of OsbHLH35-MBP and OsNAC46-MBP proteins to FAM-labeled *ProOsPT1$^{H\text{-}MITE}$* probe. MBP serves as a negative control.

(B) EMSAs demonstrating direct binding of OsbHLH35 to the H-*MITE* sequence. Competitor probes were added at 2- and 20-fold excess.

(C) Schematic representation of predicted OsbHLH35 binding sites (BS1–BS6) on *ProOsPT1$^{H\text{-}MITE}$* and in *ProOsPT1.*

(D) EMSAs showing that OsbHLH35 can bind to BS1 and BS2 (located in the H-*MITE*).

(E) EMSAs showing that OsbHLH35 can bind to BS3, BS5, and BS6.

(F to H) EMSAs to confirm the binding of OsbHLH35 to the three binding sites in the *OsPT1$^{H\text{-}MITE}$* promoter. The competitor probe and mutant probe were added at 50-, 100-, and 200-fold molar excess of labeled probes, respectively.

(I) Yeast one-hybrid showing direct binding of OsbHLH35 to the *OsPT1$^{H\text{-}MITE}$* promoter.

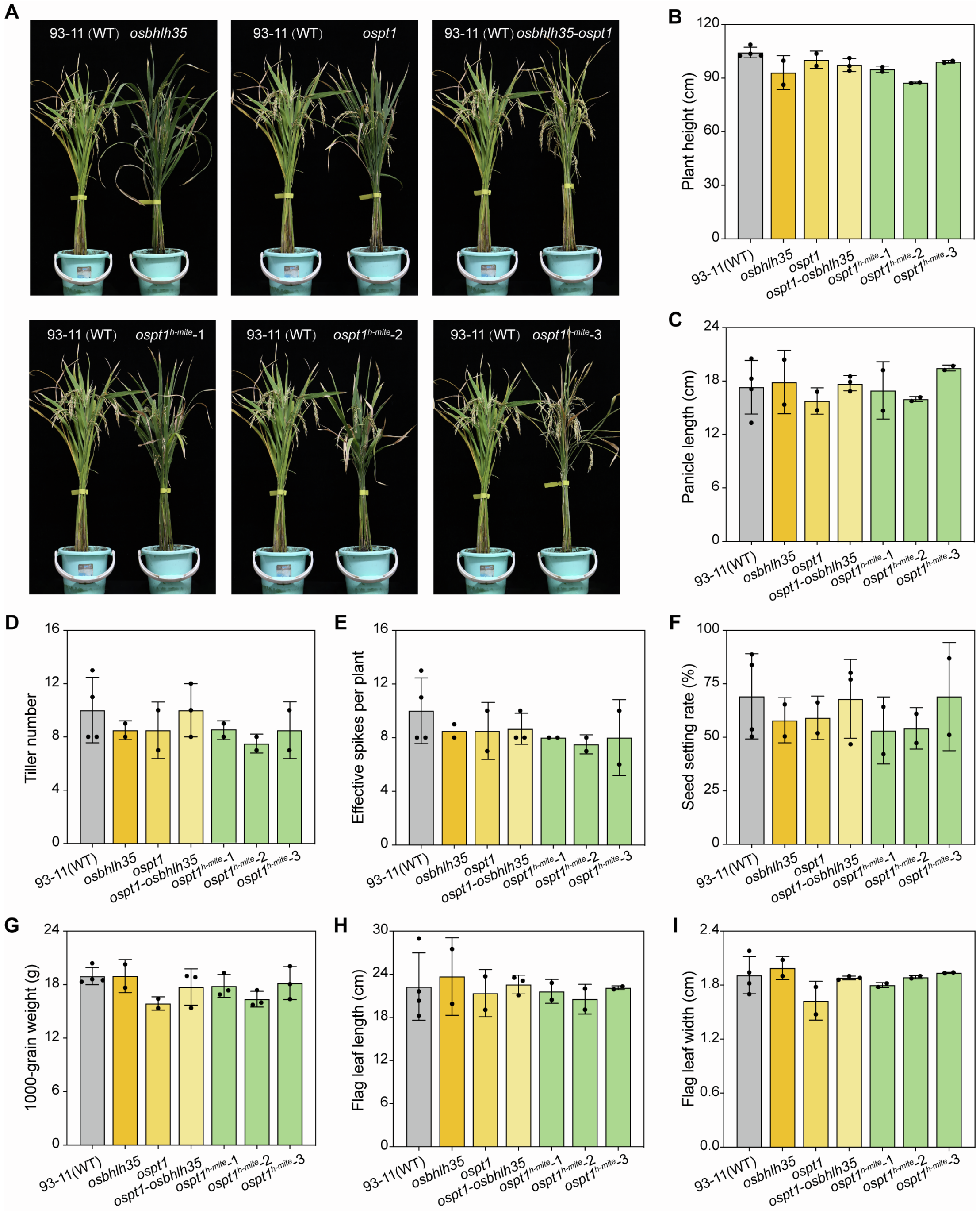

Figure S9. The agronomic traits were evaluated in *ospt1*, *osbhlh35*, and *ospt1*$^{h\text{-}mite}$ knockout mutant plants.

(A) Phenotypes of wild type 93-11, *osbhlh35*, *ospt1*, *osbhlh35-ospt1,* and *ospt1*$^{h\text{-}mite}$ mutant plants.

(B to I) The agronomic traits of wild type, *ospt1, osbhlh35, ospt1-osbhlh35,* and *ospt1*$^{h\text{-}mite}$ mutant plants. Plant height **(B)**, panicle length **(C)**, tiller number **(D)**, effective spikes per plant **(E)**, seed setting rate **(F)**, 1000-grain weight **(G)**, flag leaf length **(H)**, and flag leaf width **(I)**. Error bars represent SD; asterisks denote significant differences according to Student's *t*-test (**P* < 0.05, ***P* < 0.01).

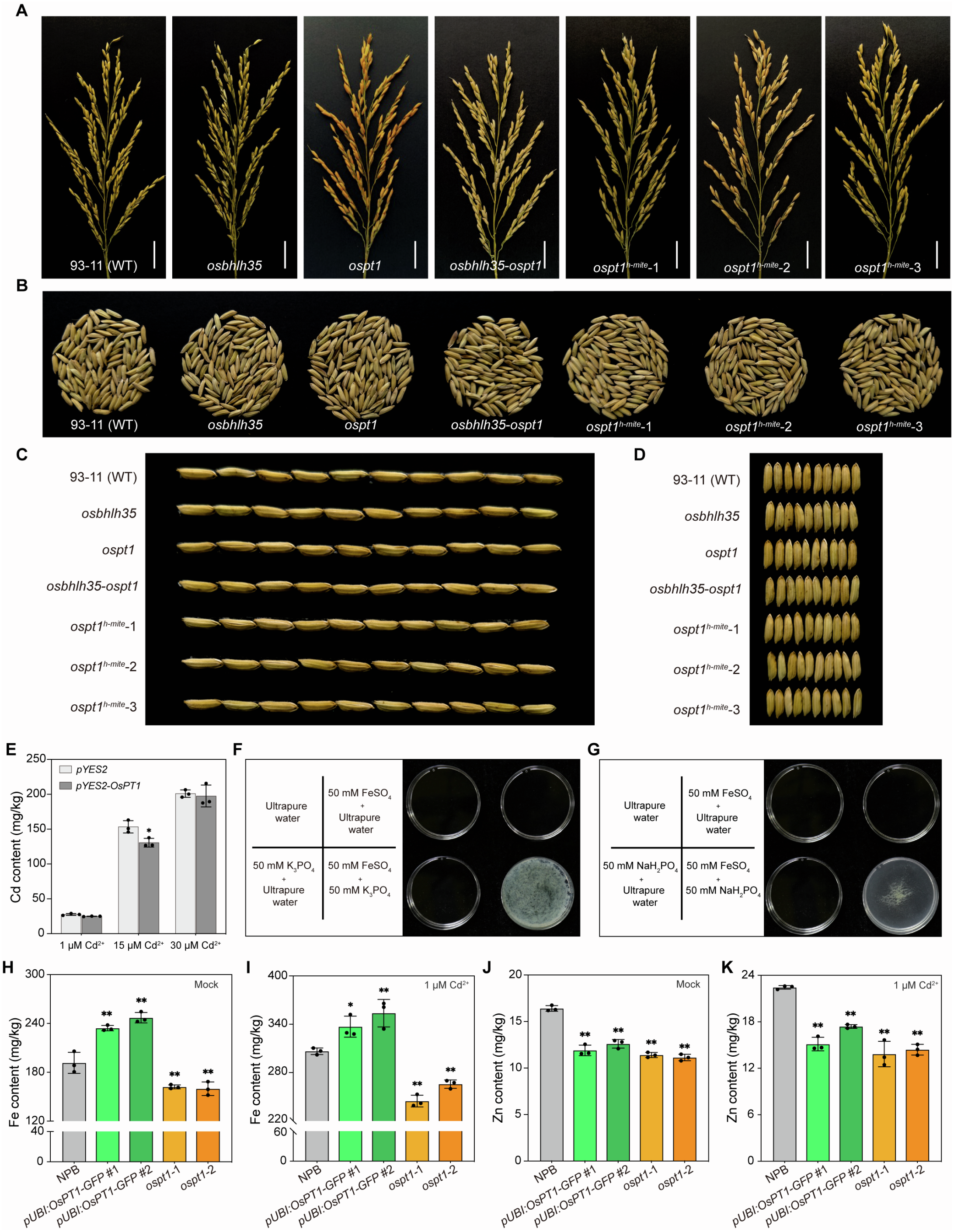

Figure S10. Phenotypes of *osbhlh35*, *ospt1*, and *in vitro* assay to test the interaction between phosphate and different ions.

(A to D) Representative seed phenotypes of wild-type 93-11, *osbhlh35*, *ospt1*, *osbhlh35-ospt1,* and *ospt1*$^{h\text{-}mite}$ mutants' plants. Scale bars = 5 cm.

(E) Cd content in wild type yeast strain *INVSc1* (empty vector: *pYES2*) and *INVSc1* expressing *OsPT1* (*pYES2-OsPT1*) after being treated with Cd^{2+} for 24 hours. Error bars represent SD; asterisks denote significant differences according to Student's *t*-test (*P < 0.05, **P < 0.01).

(F and G) In *vitro* assay to test the interaction between phosphate and Fe^{2+}.

Both $(PO_4)^{3-}$ (F) and $(H_2PO_4)^-$ (G) can strongly bind to Fe^{2+} and quickly react to form the precipitate within 5 minutes.

(H and I) Fe content of the rice shoots under normal **(H)** and 1 μM Cd^{2+} stress **(I)** conditions in *OsPT1* overexpression and *ospt1* mutant plants. The error bars represent SD; the asterisks denote significant differences according to Student's *t*-test (*$P < 0.05$, **$P < 0.01$).

(J and K) Zn contents of the rice shoots under normal **(J)** and 1 μM Cd^{2+} stress **(K)** conditions in *OsPT1* overexpression and *ospt1* mutant plants. Error bars represent SD; asterisks denote significant differences according to Student's *t*-test (*$P < 0.05$, **$P < 0.01$).
