# Supplementary material for: A transposon insertion in the 5′ UTR of OsPT1 reprograms its expression pattern and promotes cadmium accumulation in rice grains
Source: Plant Commun. 2025 Oct 15;7(1):101566. doi: 10.1016/j.xplc.2025.101566 (PMC12902297; doi:10.1016/j.xplc.2025.101566)
Supplement: Document S2. Article plus supplemental information [file mmc3.pdf]

Plant Communications
Research article

CellPress
Partner Journal

A transposon insertion in the 5′ UTR of *OsPT1* reprograms its expression pattern and promotes cadmium accumulation in rice grains

Shasha Peng[1,2,8], Dan Wang[2,8], Jinling Liu[2,8], Su Jiang[1,2], Yuchen Xu[1,2], Yufei Deng[1,2], Xiaolong Zhou[2], Fangzhi Hu[2], Zhuo Liu[2], Ye Peng[1], Hejun Ao[2], Yinghui Xiao[2], Jiurong Wang[3], Junliang Zhao[4], Bin Liu[4], Keke Yi[5], Lianyang Bai[2,6,*], Guo-Liang Wang[7,*] and Houxiang Kang[1,*]

[1]State Key Laboratory for Biology of Plant Diseases and Insect Pests, Institute of Plant Protection, Chinese Academy of Agricultural Sciences, Beijing 100193, China

[2]Hunan Provincial Key Laboratory of Crop Germplasm Innovation and Utilization and College of Agronomy, Hunan Agricultural University, Changsha, Hunan 410128, China

[3]Key Laboratory of Agro-Ecological Processes in Subtropical Region, Institute of Subtropical Agriculture, Chinese Academy of Sciences, Changsha 410125, China

[4]Guangdong Key Laboratory of New Technology in Rice Breeding, Rice Research Institute, Guangdong Academy of Agricultural Sciences, Guangzhou 510640, China

[5]Institute of Agricultural Resources and Regional Planning, Chinese Academy of Agricultural Sciences, Beijing 100081, China

[6]Hunan Weed Science Key Laboratory, Hunan Academy of Agricultural Sciences, Changsha 410125, China

[7]Department of Plant Pathology, Ohio State University, Columbus, OH 43210, USA

[8]These authors contributed equally to this article.

***Correspondence: Lianyang Bai (lybai@hunaas.cn), Guo-Liang Wang (wang.620@osu.edu), Houxiang Kang (kanghouxiang@caas.cn)**

https://doi.org/10.1016/j.xplc.2025.101566

ABSTRACT

Cadmium (Cd) accumulation in rice grains presents a serious risk to human health; however, the mechanisms underlying this process remain incompletely understood. In this study, a genome-wide association analysis identified 29 loci associated with grain Cd content (LAGCCs). Among these, one of the most strongly associated loci, LAGCC4, contains the transporter gene *OsPT1*, whose haplotypes show a strong correlation with Cd content in rice grains. A transposon, H-*MITE*, inserts into the 5′ untranslated region (UTR) of *OsPT1*, altering its expression pattern and leading to increased Cd accumulation. Furthermore, we identified the transcription factor OsbHLH35, which specifically binds to the *OsPT1*$^{H\text{-}MITE}$ promoter to regulate its transcription in response to Cd stress. Targeted knockout of either *OsPT1*$^{H\text{-}MITE}$ or *OsbHLH35* via CRISPR-Cas9 gene editing significantly reduced grain Cd content, with reductions ranging from 61.7% to 80.6%. This study reveals a previously unrecognized mechanism contributing to high Cd accumulation in rice and identifies genetic targets for breeding rice varieties with reduced Cd content.

Keywords: rice, cadmium accumulation, loci associated with grain cadmium content, transporter, GWAS, miniature inverted-repeat transposable element

Peng S., Wang D., Liu J., Jiang S., Xu Y., Deng Y., Zhou X., Hu F., Liu Z., Peng Y., Ao H., Xiao Y., Wang J., Zhao J., Liu B., Yi K., Bai L., Wang G.-L., and Kang H. (2026). A transposon insertion in the 5′ UTR of *OsPT1* reprograms its expression pattern and promotes cadmium accumulation in rice grains. Plant Comm. **7**, 101566.

INTRODUCTION

Cadmium (Cd) is a non-essential element that poses serious risks to both plants and animals (Ma et al., 2021). Industrial and mining activities, combined with increased sewage irrigation and the extensive use of chemical fertilizers, have resulted in widespread Cd contamination of agricultural soils (Gao et al., 2022). Rice (*Oryza sativa*), a staple food for nearly half the global population, readily absorbs Cd from the soil and accumulates this toxic metal in multiple tissues, particularly in the edible grains. This accumulation serves as a major pathway for Cd entry into the human food chain (Huang et al., 2024b). The well-documented itai-itai disease arose from chronic ingestion of Cd-contaminated rice grains (Aoshima, 2017). In China, more than 10% of commercially available rice grains are estimated to contain high Cd levels (≥ 0.2 mg/kg) (Huang et al., 2024a; Zhao et al., 2024), suggesting that approximately 84.7 million people in China alone are directly affected by long-term exposure through rice consumption. Although comparable data from other countries remain limited, reducing Cd accumulation

© 2025 The Authors. Published by Elsevier Inc. on behalf of CAS Center for Excellence in Molecular Plant Sciences, Chinese Academy of Sciences, Chinese Society for Plant Biology.
This is an open access article under the CC BY license (http://creativecommons.org/licenses/by/4.0/).

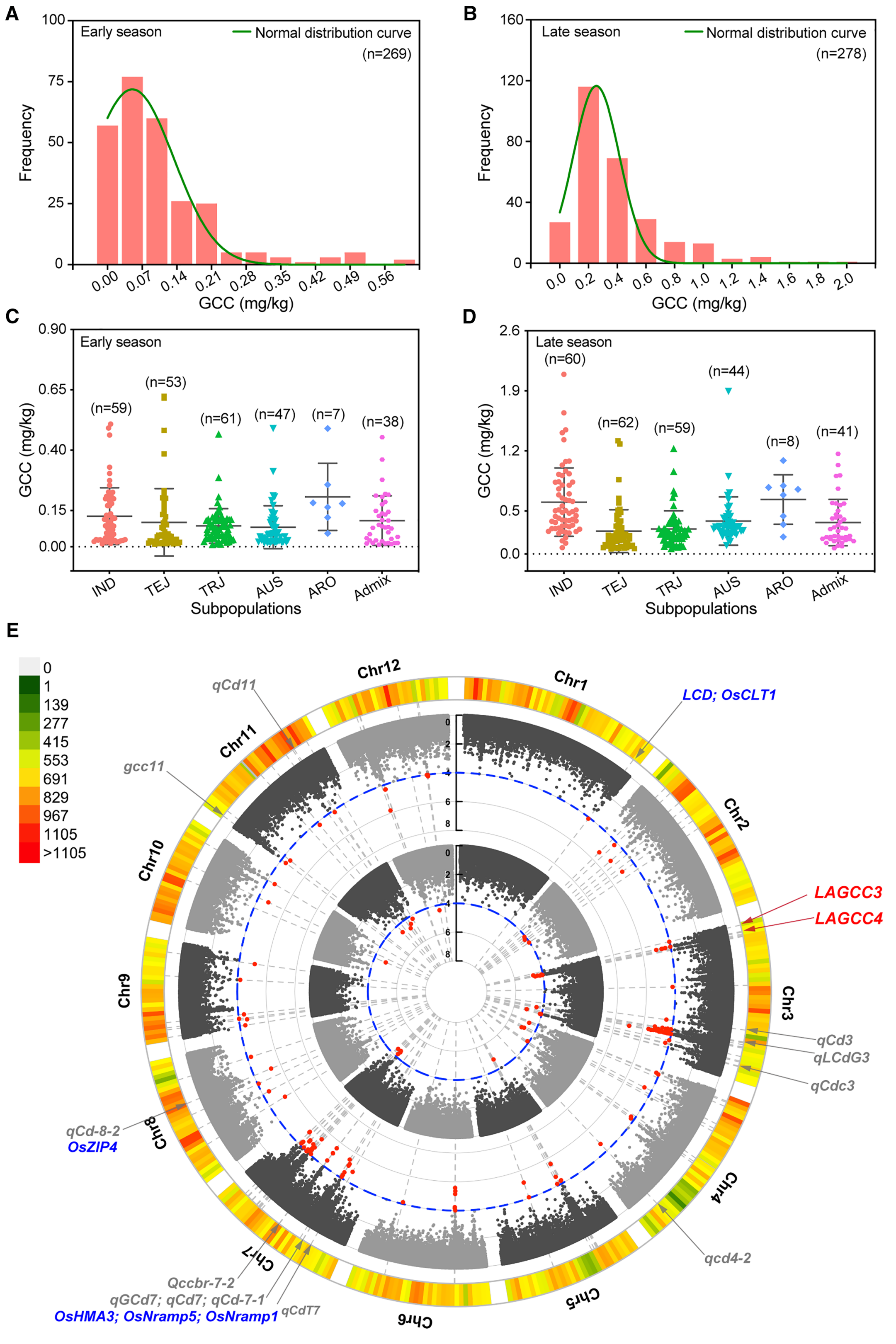

(legend on next page)

in rice grains is an urgent agricultural goal to safeguard food safety and public health.

Cd is absorbed from the soil by plant roots, transported to shoots and leaves through the vascular system, and subsequently accumulates in stems, leaves, and grains (Clemens and Ma, 2016; Zhao et al., 2022). To alleviate Cd contamination of rice grains, several strategies have been applied in rice cultivation, such as paddy water management and soil phytoremediation (Honma et al., 2016). However, these approaches are both time-consuming and costly. Notably, substantial variation in grain Cd content (GCC) among rice cultivars suggests the feasibility of selecting and breeding low-GCC varieties (Sun et al., 2016). Consequently, breeding rice cultivars with reduced Cd accumulation has become the most effective and sustainable strategy to ensure food safety.

Over the past two decades, dozens of GCC-associated genes have been cloned (Zhao et al., 2022; Sun et al., 2023). These genes can be categorized into three groups. The first group comprises proteins that mediate Cd uptake, such as IRON-REGULATED TRANSPORTER 1 (OsIRT1), which promotes Cd accumulation in roots (Nakanishi et al., 2006), and members of the natural resistance–associated macrophage protein (NRAMP) family, including OsNRAMP1 (Takahashi et al., 2011) and OsNRAMP5 (Ishikawa et al., 2012; Yu et al., 2022). Overexpression of *OsNRAMP1* increases Cd concentrations in shoots (Takahashi et al., 2011). *OsNRAMP5* transports both manganese (Mn) and Cd; loss of *OsNRAMP5* function reduces Cd uptake by approximately 90% compared with wild-type plants (Ishikawa et al., 2012; Hu et al., 2024). The second group influences root-to-shoot Cd translocation through xylem loading. The tonoplast-localized heavy metal ATPase OsHMA3 sequesters Cd into root cell vacuoles, restricting its translocation (Ueno et al., 2010; Kumagai et al., 2014). The *oshma3* mutant displays enhanced Cd transport from roots to shoots, whereas *OsHMA3* overexpression suppresses this process (Sasaki et al., 2014). The third group regulates Cd redistribution at stem nodes and remobilization from leaves to grains via the phloem (Yamaji and Ma, 2017). For instance, low-affinity cation transporter 1 (OsLCT1) functions as a phloem Cd transporter to facilitate Cd movement from enlarged to diffuse vascular bundles (Uraguchi et al., 2011). Despite these insights, the molecular mechanisms controlling Cd accumulation in rice grains remain only partly understood.

Genome-wide association studies (GWASs) have become a powerful tool to characterize genetic variation underlying complex traits and to identify candidate genomic regions in rice and other plant species (Huang et al., 2010; Zhao et al., 2011). In this study, we quantified GCC across all rice cultivars in the Rice Diversity Panel 1 (RDP1) (Eizenga et al., 2014) and identified 29 loci associated with GCC (LAGCCs) through GWASs. Of these loci, 11 co-localized with previously reported GCC-associated genes. At LAGCC4, we discovered that insertion of a Harbinger-type miniature inverted-repeat transposable element (H-*MITE*) into the 5′ untranslated region (UTR) of the transporter gene *OsPT1* alters its structure, resulting in elevated *OsPT1* expression under Cd stress. This enhanced expression disrupts cellular ion homeostasis and substantially increases GCC. Furthermore, we identified the transcription factor OsbHLH35, which specifically binds to the $OsPT1^{H\text{-}MITE}$ promoter to regulate its transcription in response to Cd stress. Targeted knockout of the H-*MITE*, *OsPT1*, or *OsbHLH35* genes significantly reduced GCC. Collectively, these findings elucidate the genetic basis of Cd accumulation in rice and reveal a previously unrecognized mechanism driving high Cd accumulation, offering valuable targets for breeding rice varieties with reduced Cd content.

RESULTS

Variation in rice grain Cd content (GCC)

A total of 310 rice cultivars from the RDP1 were grown in a Cd-contaminated field, and GCC was measured for 269 cultivars in the early season and 278 in the late season (Supplemental Table 1). GCC followed a skewed normal distribution, ranging from 0.008 to 0.454 mg/kg in early-season rice and from 0. 058 to 2.092 mg/kg in late-season rice (Figures 1A and 1B; Supplemental Table 1). Late-season *indica* (IND) accessions displayed the highest average GCC (0.604 ± 0.398 mg/kg), whereas the *temperate japonica* (TEJ) and *tropical japonica* (TRJ) subpopulations showed comparatively lower average GCC values (Figures 1C and 1D; Supplemental Table 1). These results are consistent with earlier reports indicating significantly higher GCC in IND than in TEJ and TRJ cultivars (Tan et al., 2020). Correlation analysis revealed no significant association between GCC and growth duration (Supplemental Figure 1A–1C). Furthermore, Cd exposure substantially affected multiple agronomic traits, including tiller number, under the experimental conditions (Supplemental Figure 1D).

GWAS identifies 29 loci associated with GCC (LAGCCs)

To investigate the genetic basis of GCC, 700 000 single-nucleotide polymorphisms (SNPs) from the RDP1 were analyzed for associations with Cd accumulation. A GWAS identified 157 SNPs significantly associated with GCC (Supplemental Table 2).

Figure 1. Variation in rice grain Cd contents (GCC) within the RDP1.
(A and B) Distribution of GCC in early-season rice **(A)** and late-season rice **(B)**.
(C and D) Scatterplots illustrating GCC variation among rice subpopulations in early-season rice **(C)** and late-season rice **(D)**. Each dot represents an accession. Horizontal lines denote mean GCC, and error bars indicate standard deviation (SD). IND, *indica*; TEJ, temperate *japonica*; TRJ, tropical *japonica*; AUS, *aus*; ARO, *aromatic*; Admix, admixed.
(E) Circular Manhattan plot displaying the genomic locations of 29 loci associated with GCC (LAGCCs). The inner circle represents the Manhattan plot for early-season rice, and the outer circle corresponds to late-season rice. Each dot represents an SNP, and the black scale indicates $-\log_{10}(P \text{ value})$. SNPs exceeding the significance threshold of $-\log_{10}(P \text{ value}) = 4$ (blue dashed circle) are highlighted in red. The outermost ring shows SNP density across the 12 rice chromosomes. Previously mapped or cloned QTLs for grain Cd accumulation are shown in gray and corresponding candidate genes in blue. Red arrows indicate the focal regions LAGCC3 and LAGCC4 emphasized in this study.
See also Supplemental Figure 1 and Supplemental Tables 1–3.

Based on the average size of linkage disequilibrium (LD) decay blocks in rice (Mather et al., 2007), these SNPs were clustered into 29 non-redundant LAGCCs, collectively explaining 58.6% (P = 0.04) and 45.8% (P = 0.05) of the phenotypic variation in GCC in the early- and late-season RDP1 populations, respectively. Among the 29 LAGCCs, eight loci were consistently identified across both growing seasons, and 11 co-localized with previously cloned genes or mapped quantitative trait loci (QTLs) associated with Cd accumulation (Figure 1E; Supplemental Table 3). For instance, LAGCC1 overlapped with *Low Cadmium* (*LCD*) (Shimo et al., 2011) and *OsCLT1* (Zhong et al., 2024), LAGCC16 overlapped with *OsNRAMP5* (Ishikawa et al., 2012) and *OsNRAMP1* (Takahashi et al., 2011), and LAGCC5 contained *qCd3* (Norton et al., 2009). Eighteen loci represented newly identified LAGCCs reported in this study (Supplemental Table 3).

The transporter gene *OsPT1* is associated with Cd content (GCC)

LAGCC3 and LAGCC4, both located on rice chromosome 3, did not overlap with previously reported GCC-associated genes, prompting their selection for further investigation (Figure 1E). Given that most known GCC-associated genes encode ion transporters (Nakanishi et al., 2006; Takahashi et al., 2011; Sasaki et al., 2012; Zulfiqar et al., 2022), the LAGCC3 and LAGCC4 regions were examined for transporter-encoding genes. This analysis identified Os03g04920, Os03g05290, Os03g05620, Os03g06080, and Os03g06139 (Supplemental Table 4). The genomic regions of these five genes were cloned and sequenced in 10 high-GCC and 16 low-GCC accessions (Supplemental Table 5). Across the 26 rice accessions, 53 polymorphisms were detected in Os03g05290 and 28 in Os03g05620 (Supplemental Figure 2A and 2B). None of the polymorphisms in Os03g05290 was associated with GCC. However, six polymorphisms within the promoter region of Os03g05620 (hereafter referred to as *OsPT1*) (Sun et al., 2012) were strongly associated with GCC (Figure 2A and Supplemental Figure 3A). *OsPT1* is located between SNP 3.2705191 and SNP 3.2966886, spanning genomic coordinates 2 706 196–2 967 891 on chromosome 3 (Supplemental Table 4). *OsPT1* encodes a phosphate transporter (Seo et al., 2008; Sun et al., 2012) (Supplemental Figure 2E), and its plasma membrane localization was confirmed (Figures 2B–2D and Supplemental Figure 3B and 3C). Further sequence analysis revealed a 266-bp insertion located 354 bp upstream of the *OsPT1* transcription start site (TSS) in the 93-11 rice cultivar but absent in *Nipponbare* (NPB) (Figure 2A and Supplemental Figures 2B and 3A). Haplotype analysis of *OsPT1* in RDP1, based on the 266-bp insertion and two GCC-associated SNPs, grouped the accessions into two major haplotypes: haplotype A (lacking the 266-bp insertion, comprising A-1, A-2, and A-3; Figure 2E) and haplotype B (containing the 266-bp insertion, comprising B-1, B-2, and B-3; Figure 2E). Accessions with haplotype B exhibited a median GCC of 0.24 mg/kg, significantly higher than the 0.13 mg/kg median observed in haplotype A accessions (Wilcoxon rank-sum test, $P = 9.6 \times 10^{-6}$), indicating a robust haplotype effect on Cd accumulation (Figure 2F; Supplemental Table 6). Across subpopulations, 75.3% of *japonica* accessions carried haplotype A-1, whereas 86.7% of IND accessions displayed haplotype B-3 (Figure 2G). These results are consistent with previous reports documenting relatively high GCC in IND and low GCC in *japonica* rice (Song et al., 2015).

To further validate the association between *OsPT1* genotype and GCC, a multi-year field evaluation was performed using 12 rice accessions (six with haplotype A-1 and six with haplotype B-3) cultivated in the same Cd-contaminated field over 3 years (2016, 2017, and 2019) (Supplemental Table 6). Statistically significant differences in average GCC were observed between accessions carrying haplotypes A-1 and B-3 across all 3 years (P = 0.0005, 0.0001, and 0.0042 for 2016, 2017, and 2019, respectively) (Figures 2H–2J). These results demonstrate the temporal stability of this genotype–phenotype association under uniform environmental conditions.

Analysis of the complete RDP1 population revealed similar patterns of Cd accumulation between haplotypes across both subspecies. Among 16 IND accessions, five haplotype A accessions (lacking the 266-bp insertion) exhibited a relatively low average GCC (0.244 ± 0.063 mg/kg), whereas 55 haplotype B accessions (carrying the insertion) showed significantly higher average GCC (0.648 ± 0.396 mg/kg). Similarly, among 121 *japonica* accessions, 101 haplotype A accessions displayed a relatively low average GCC (0.233 ± 0.171 mg/kg), whereas 20 haplotype B accessions with the insertion exhibited a higher average GCC (0.480 ± 0.294 mg/kg) (Supplemental Figure 4A; Supplemental Table 7). These comprehensive analyses confirm that the 266-bp H-*MITE* insertion in *OsPT1* is consistently associated with increased Cd accumulation in rice grains across both IND and japonica subspecies.

The 266-bp sequence is a *MITE* that shows insertion polymorphisms in 3K-RG and RDP1

Sequence analysis identified the 266-bp insertion as an H-*MITE* (Figure 3A). To examine its distribution across diverse rice accessions, we analyzed an independent rice population from the 3000 Rice Genomes Project (3K-RG) (Wang et al., 2018), selecting 430 accessions with sequencing coverage exceeding 20× that did not overlap with RDP1 accessions (Supplemental Table 8). Using established methods to detect transposable element (TE; transposon) insertion polymorphisms (Kang et al., 2016), we found that 70.7% (304 of 430) of the accessions carried the H-*MITE* insertion. The insertion was nearly ubiquitous in IND accessions, present in 98.3% (284 of 289), but rare in *japonica* accessions, detected in only 2.2% (2 of 92). These findings are consistent with those observed in the RDP1 population.

To further elucidate the genetic architecture underlying Cd accumulation, we performed genotype analyses of four known Cd transporter genes—*OsNRAMP1* (Takahashi et al., 2011), *OsNRAMP5* (Ishikawa et al., 2012), *OsHMA3* (Ueno et al., 2010), and *OsCd1* (Yan et al., 2019)—in 26 accessions exhibiting contrasting GCC phenotypes (Supplemental Table 5). All four genes displayed robust genotype–phenotype associations with GCC (Supplemental Figure 4B–4E), indicating that Cd accumulation is influenced by multiple genetic factors.

Geographic analysis of rice varieties from both the 3K-RG and RDP1 populations revealed distinct regional distribution patterns

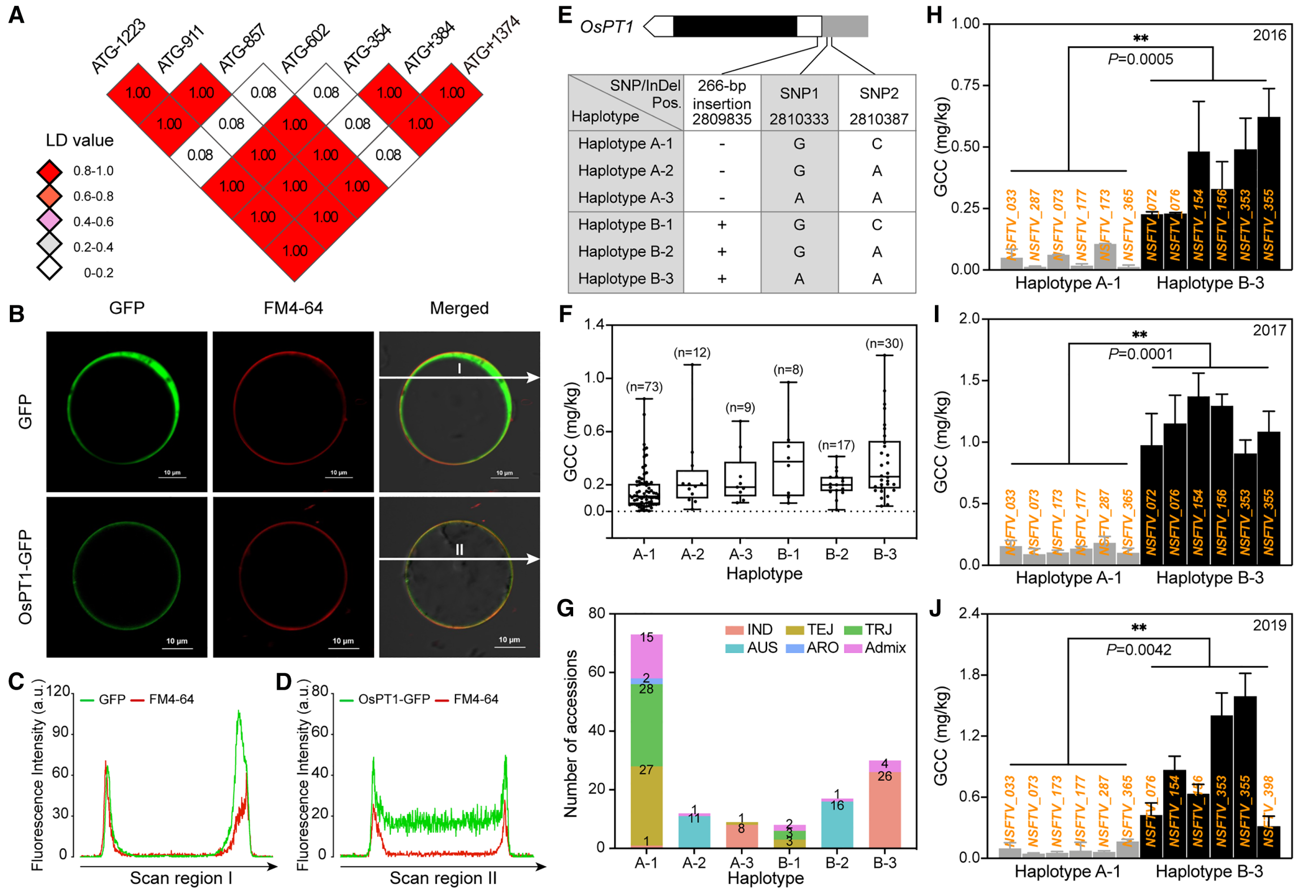

Figure 2. Haplotype analysis and linkage disequilibrium (LD) heatmap of *OsPT1*.

(A) LD heatmap illustrating pairwise LD values for markers within *OsPT1*.

(B–D) Confocal fluorescence microscopy images showing the subcellular localization of OsPT1 **(B)** and merged fluorescence signals **(C and D)** in rice protoplasts expressing GFP and OsPT1-GFP with FM4-64 staining.

(E) The *OsPT1* locus contains two major haplotypes. Accessions carrying haplotype A lack the 266-bp insertion in the *OsPT1* 5′ UTR; all haplotype B accessions carry the 266-bp insertion.

(F) Average GCC among the *OsPT1* haplotypes. Each dot represents a rice accession. Statistical significance was determined using the Wilcoxon rank-sum test ($P = 9.6 \times 10^{-6}$).

(G) Distribution of *OsPT1* haplotypes across rice subpopulations. Most accessions with haplotype A-1 are *japonica* rice, whereas most accessions with haplotype B-3 are IND rice. **(H–J)** Average GCC of six accessions with haplotype A-1 and six accessions with haplotype B-3 in 2016 **(H)**, 2017 **(I)**, and 2019 **(J)**.

Error bars represent SD; asterisks denote significant differences according to Student's *t*-test (*$P < 0.05$, **$P < 0.01$). See also Supplemental Figures 2–4 and Supplemental Tables 4–7.

(Figure 3B and 3C). Haplotype A, characterized by the absence of the H-*MITE* insertion, is predominant in regions such as the United States, Japan, Europe, and several African countries. Conversely, haplotype B, which carries the H-*MITE* insertion, is primarily found in major rice-producing regions, including China, India, and Southeast Asian countries. This distribution pattern suggests that rice varieties cultivated in many emerging countries have an increased tendency for elevated Cd accumulation, underscoring food safety concerns in these areas.

The H-*MITE* insertion alters the *OsPT1* gene structure and expression pattern

To assess the effect of the H-*MITE* insertion on the *OsPT1* gene structure, 5′ rapid amplification of cDNA ends (RACE) experiments were conducted to identify the TSS in the rice accessions NPB (lacking H-*MITE* insertion in *OsPT1*) and 93-11 (containing H-*MITE* insertion in *OsPT1*). The TSS in NPB was located 443 bp upstream of the translation start codon (Figure 3A). In contrast, the H-*MITE* insertion disrupted the 5′ UTR of *OsPT1* in 93-11, generating a novel 408-bp 5′ UTR composed of a 54-bp H-*MITE* fragment and a 354-bp segment from the original 5′ UTR. These findings indicate that the H-*MITE* insertion modifies the 5′ UTR and TSS of *OsPT1* without affecting the translation start codon.

Considering the critical role of the 5′ UTR in mRNA stability and translation efficiency (Jia et al., 2020), we hypothesized that the H-*MITE*–mediated modification of the *OsPT1* 5′ UTR might influence gene expression at the transcriptional or translational level. To test this hypothesis, OsPT1 transcript levels were quantified by reverse transcription (RT)–quantitative polymerase chain reaction (qPCR) in rice accessions with (93-11) or without (NPB) the

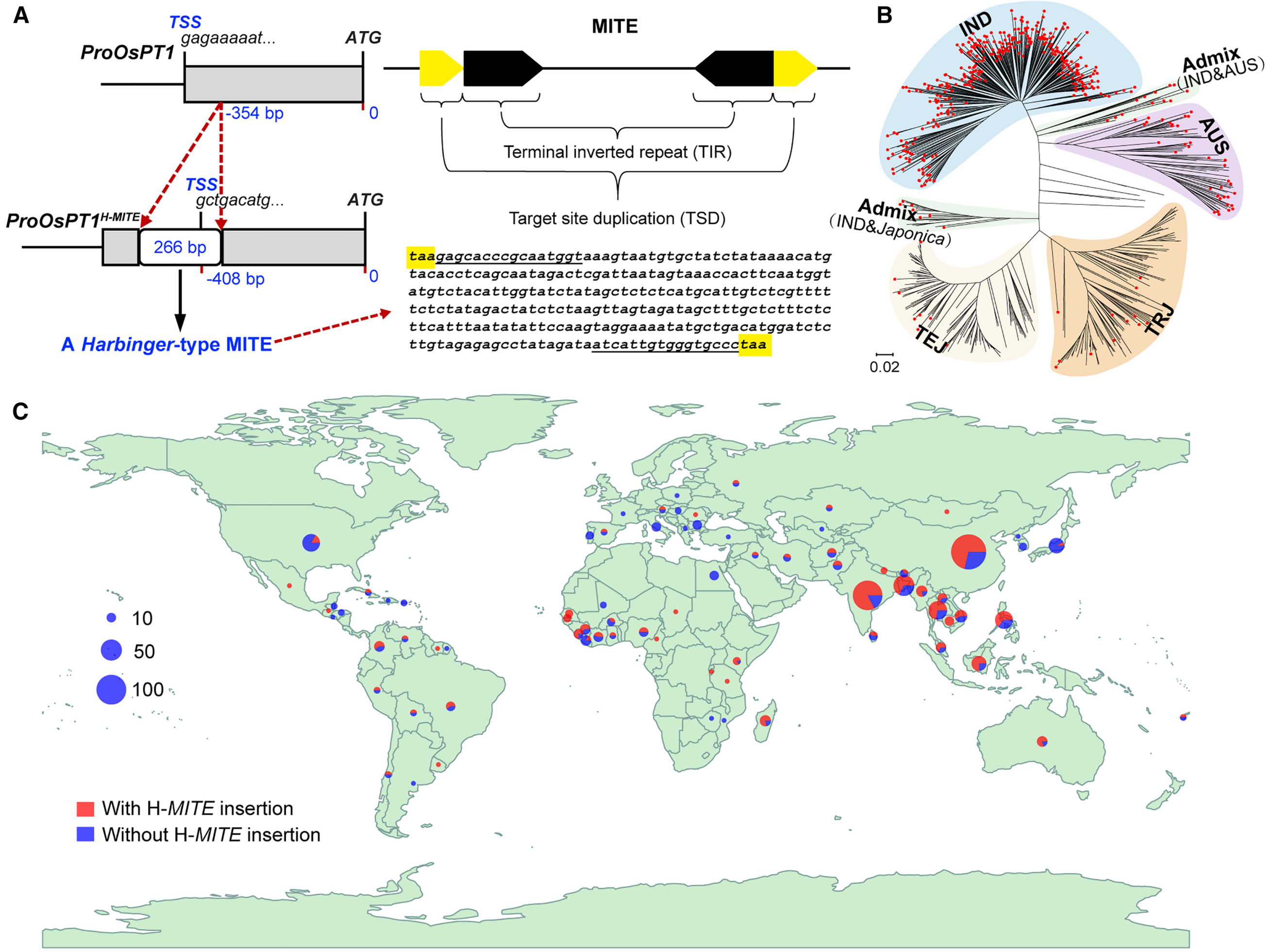

Figure 3. A *Harbinger*-type miniature inverted-repeat transposable element (H-*MITE*) insertion in the 5′ UTR of *OsPT1* is widespread in *indica* rice.

(A) Schematic diagram showing alteration of the transcription start site (TSS) of *OsPT1* caused by a 266-bp H-*MITE* insertion in the 5′ UTR (left) and the structure and sequence of the H-*MITE* element (right). Yellow boxes denote target site duplication (TSD) sequences, and underlined sequences indicate terminal inverted repeats (TIRs).

(B) Neighbor-joining phylogenetic tree of rice accessions from 3K-RG and RDP1. Each branch corresponds to a rice accession. Red dots represent accessions carrying the H-*MITE* insertion. Rice subpopulations are color-coded.

(C) Global distribution of rice varieties with and without the H-*MITE* insertion across major rice-producing countries. The size of each symbol is proportional to the number of accessions.

See also Supplemental Figure 4 and Supplemental Table 8.

H-*MITE* insertion. Under normal growth conditions, OsPT1 transcript abundance was lower in 93-11 than in NPB. However, after exposure to 1 and 5 μM Cd^{2+} stress, *OsPT1* expression was strongly induced in 93-11 but remained unchanged in NPB (Figures 4A and 4B). These results suggest that the H-*MITE* insertion within the 5′ UTR of *OsPT1* reprograms its expression profile under both normal and Cd stress conditions.

A promoter activity assay was conducted to compare the activities of two *OsPT1* promoter variants: *ProOsPT1*, amplified from NPB lacking the H-*MITE* insertion, and *ProOsPT1*$^{H\text{-}MITE}$, amplified from 93-11 containing the H-*MITE* insertion. Each promoter fragment was cloned upstream of the β-glucuronidase (GUS) reporter gene, and the constructs were used to generate stable transgenic rice plants. GUS staining revealed that *ProOsPT1*$^{H\text{-}MITE}$, but not *ProOsPT1*, exhibited prominent upregulation in multiple rice tissues under 5 μM Cd^{2+} stress (Figures 4C–4E and Supplemental Figure 5A and 5B). This finding constitutes further evidence that the H-*MITE* insertion modifies the expression pattern of *OsPT1* under Cd stress.

Taken together, our results indicate that the H-*MITE* insertion restructures the *OsPT1* promoter and confers Cd-inducible expression, revealing a novel mechanism through which TEs can reprogram gene-regulatory networks in response to environmental stress.

The H-*MITE* insertion at the *OsPT1* promoter leads to high Cd accumulation in rice

To investigate the role of *OsPT1* in rice grain Cd accumulation, we generated *OsPT1* overexpression (*pUBI:OsPT1-GFP*) transgenic

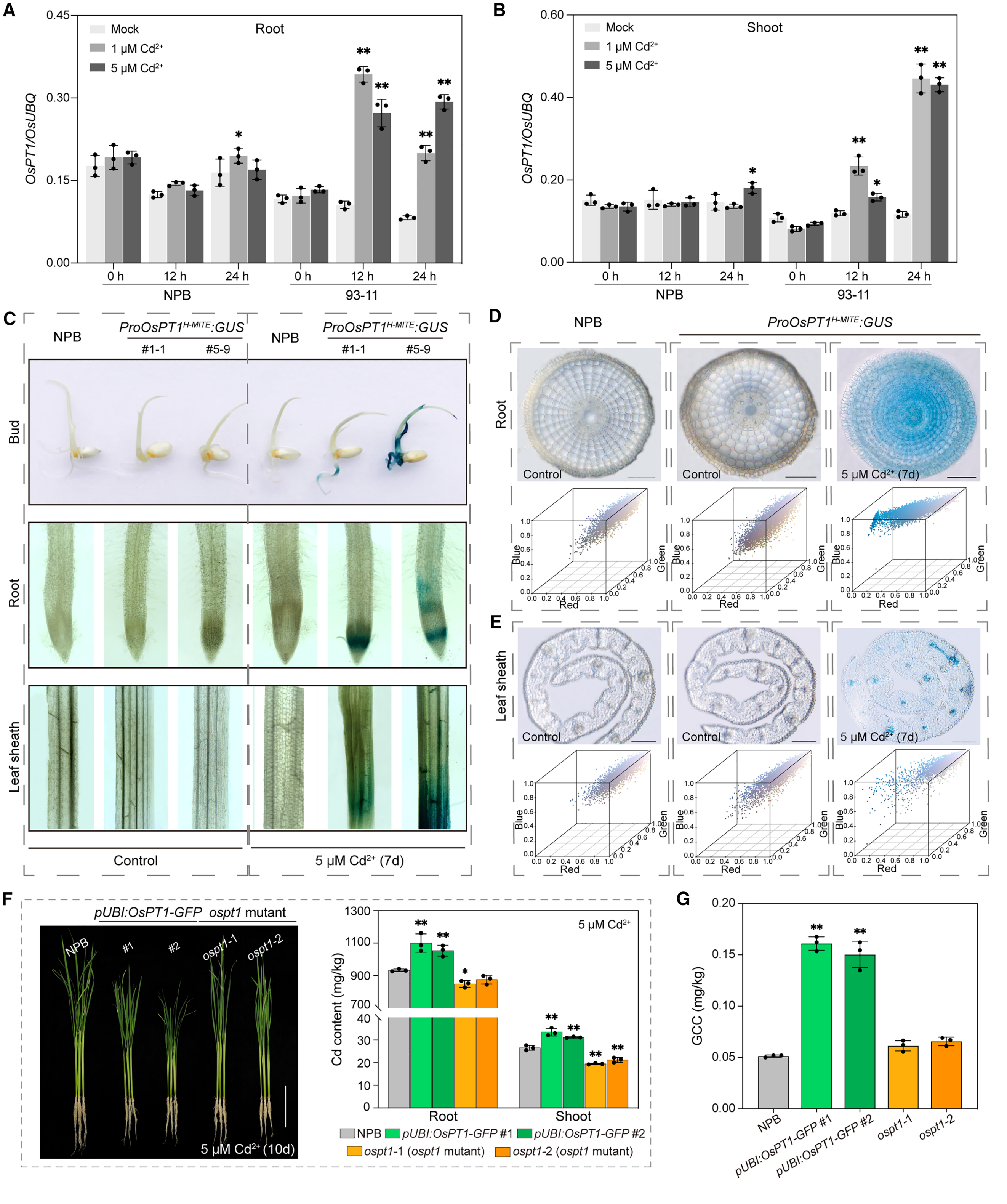

Figure 4. Cd treatment induces *GUS* transcription in stable *ProOsPT1*$^{H\text{-}MITE}$*:GUS* transgenic plants, and overexpression of *OsPT1* causes high Cd accumulation in rice.

(A and B) Relative OsPT1 transcript levels in roots **(A)** and shoots **(B)** under Cd stress, as determined by RT–qPCR. Expression values were normalized to *OsUBQ*. NPB, *Nipponbare* (without H-*MITE* insertion in *OsPT1*); 93-11, accession *93-11* (with H-*MITE* insertion). Error bars represent SD; asterisks denote significant differences according to Student's *t*-test (*$P < 0.05$, **$P < 0.01$).

(legend continued on next page)

lines and the *ospt1* knockout mutant in the NPB background. A total of 25 overexpression and 58 mutant lines were obtained; homozygous T_2 lines were selected for subsequent analyses (Supplemental Figure 6A and 6B). In a hydroponic culture system, *OsPT1* overexpression seedlings exposed to 1 and 5 μM Cd^{2+} accumulated more Cd in roots and shoots than wild-type plants ($P < 0.0327$ in roots, $P < 0.0069$ in shoots), whereas *ospt1* mutants accumulated less Cd compared with wild-type plants ($P < 0.0416$ in roots, $P < 0.0143$ in shoots) (Figure 4F and Supplemental Figure 6C and 6D). Under Cd-contaminated soil conditions (soil Cd^{2+} concentration, 0.85 mg/kg) (Li et al., 2022), GCC was significantly higher in *OsPT1*-overexpressing plants (0.161 and 0.15 mg/kg, $P < 0.0001$) than in wild-type plants (0.045 mg/kg) (Figure 4G). These results indicate that *OsPT1* overexpression enhances Cd accumulation in both rice seedlings and grains.

Furthermore, *ospt1* mutant plants displayed normal agronomic traits—including plant height, tiller number, panicle length, and 1000-grain weight—compared with wild-type NPB. However, *OsPT1* overexpression plants exhibited dwarfism accompanied by reductions in panicle length and 1000-grain weight (Seo et al., 2008) (Supplemental Figures 2C, 2D, and 7A–7H).

The transcription factor OsbHLH35 binds to the *OsPT1$^{H\text{-}MITE}$* promoter to regulate its transcription

The H-*MITE* insertion in the *OsPT1* promoter confers Cd-inducible transcription. To identify the upstream transcription factor mediating this induction under Cd exposure, two promoter fragments—*ProOsPT1* (468 bp) and *ProOsPT1$^{H\text{-}MITE}$* (734 bp)—were used as bait in protein pull-down assays. Subsequent liquid chromatography–tandem mass spectrometry (LC–MS/MS) analysis identified 69 proteins binding exclusively to *ProOsPT1* and 59 proteins specific to *ProOsPT1$^{H\text{-}MITE}$* (Figure 5A). Among the 59 proteins uniquely interacting with *ProOsPT1$^{H\text{-}MITE}$* (Supplemental Table 9), three transcription factors were detected: OsbHLH35 (Os01g06640), OsMADS79 (Os01g74440), and OsNAC46 (Os08g33670). An electrophoretic mobility shift assay (EMSA) confirmed that OsbHLH35 and OsNAC46 directly bind to the *ProOsPT1$^{H\text{-}MITE}$* fragment *in vitro* (Supplemental Figure 8A). OsbHLH35 was selected for further functional characterization because its transcript levels were elevated under Cd stress, particularly in IND rice (Figure 5B). A previous report also indicated that OsbHLH35 is induced by Cd stress (Farooq et al., 2016), suggesting a role for this transcription factor in the Cd stress response.

EMSAs were conducted to compare OsbHLH35 binding affinity between the *ProOsPT1$^{H\text{-}MITE}$* and *ProOsPT1* promoter fragments. The results demonstrated that OsbHLH35 exhibited strong binding to *ProOsPT1$^{H\text{-}MITE}$*, whereas its binding to *ProOsPT1* was weak and readily competed by excess unlabeled probe (Figure 5C). To further characterize OsbHLH35 binding sites (BSs) within *ProOsPT1$^{H\text{-}MITE}$*, we utilized the PlantPAN online tool for transcription factor BS prediction (http://PlantPAN.itps.ncku.edu.tw) (Chow et al., 2019), identifying six candidate BSs (Supplemental Figure 8B and 8C). EMSA confirmed five of these as *bona fide* OsbHLH35 BSs (Supplemental Figure 8D–8H). Among them, BS1 (sequence: GCACCCGC) and BS2 (sequence: CCTACTTGG), located at +3 bp and −62 bp within the H-*MITE* fragment, respectively, displayed the strongest binding affinity (Figure 5D).

Next, we assessed the effect of OsbHLH35 on *OsPT1* transcriptional activity through a transcriptional regulation activity assay. Promoter fragments *ProOsPT1$^{H\text{-}MITE}$* and *ProOsPT1* were cloned upstream of the *firefly luciferase* (*LUC*) reporter gene, with OsbHLH35 co-expressed as an effector (Figure 5E). Transient expression in rice protoplasts revealed that OsbHLH35 substantially activated transcription driven by *ProOsPT1$^{H\text{-}MITE}$* but not by *ProOsPT1* (Figure 5E). Deletion of either BS1 or BS2 within *ProOsPT1$^{H\text{-}MITE}$* significantly attenuated OsbHLH35-mediated activation; deletion of both sites completely abolished this effect (Figure 5E). A yeast one-hybrid assay confirmed the specific interaction between OsbHLH35 and the *OsPT1$^{H\text{-}MITE}$* promoter region (Supplemental Figure 8I), supporting a regulatory role for OsbHLH35 in *OsPT1* expression.

Finally, *OsPT1* transcript levels were examined under 1 μM Cd^{2+} stress in two independent *OsbHLH35* knockout mutants (*osbhlh35-1* and *osbhlh35-2*) and wild-type 93-11. *OsPT1$^{H\text{-}MITE}$* expression was strongly induced in the wild type but markedly diminished in both mutants (Figure 5F), indicating that OsbHLH35 is essential for Cd-induced activation of *OsPT1$^{H\text{-}MITE}$*.

In summary, these *in vitro* and *in vivo* results demonstrate that Cd stress triggers accumulation of the transcription factor OsbHLH35, which robustly activates *OsPT1* transcription via direct binding to *ProOsPT1$^{H\text{-}MITE}$*.

Knockout of *OsPT1* and *OsbHLH35* or targeted editing of the transposon insertion site of *OsPT1$^{H\text{-}MITE}$* in 93-11 significantly decreases rice Cd content (GCC)

Because of the high Cd accumulation and presence of the H-*MITE* insertion in *OsPT1* within the IND rice cultivar 93-11, CRISPR-Cas9 technology was utilized to generate *OsPT1* and

(C) GUS staining of buds, roots, and leaf sheaths in wild-type (NPB) plants and two *ProOsPT1$^{H\text{-}MITE}$:GUS* transgenic lines. Left: control samples without Cd treatment. Right: Cd-treated samples exposed to ½-strength Murashige and Skoog medium supplemented with Cd for 5 days or seedlings treated with Cd for 7 days.

(D and E) GUS staining of roots **(D)** and leaf sheaths **(E)** visualized in vibratome sections. The two leftmost columns show controls (no Cd treatment), and the rightmost column shows Cd-treated samples. GUS activity was detected in both roots and leaf sheaths. Cubes below each micrograph display extracted red/green/blue (RGB) pixel values. Scale bars: 100 μm.

(F) Phenotypes of wild type, the *ospt1* mutant, and *OsPT1*-overexpressing (*pUBI:OsPT1-GFP*) lines. Left: two-week-old rice seedlings transferred to hydroponic growth conditions containing 5 μM Cd^{2+} for 10 days. Right: Cd content in roots and shoots after treatment with 5 μM Cd^{2+} for 10 days in *OsPT1*-overexpressing and *ospt1* mutant plants.

(G) Cd concentrations in the grains of *OsPT1*-overexpressing (*pUBI:OsPT1-GFP*) and *ospt1* mutant plants grown in soil containing 0.85 mg/kg Cd.

Error bars represent SD; asterisks denote significant differences according to Student's *t*-test (*$P < 0.05$, **$P < 0.01$). See also Supplemental Figures 5–7.

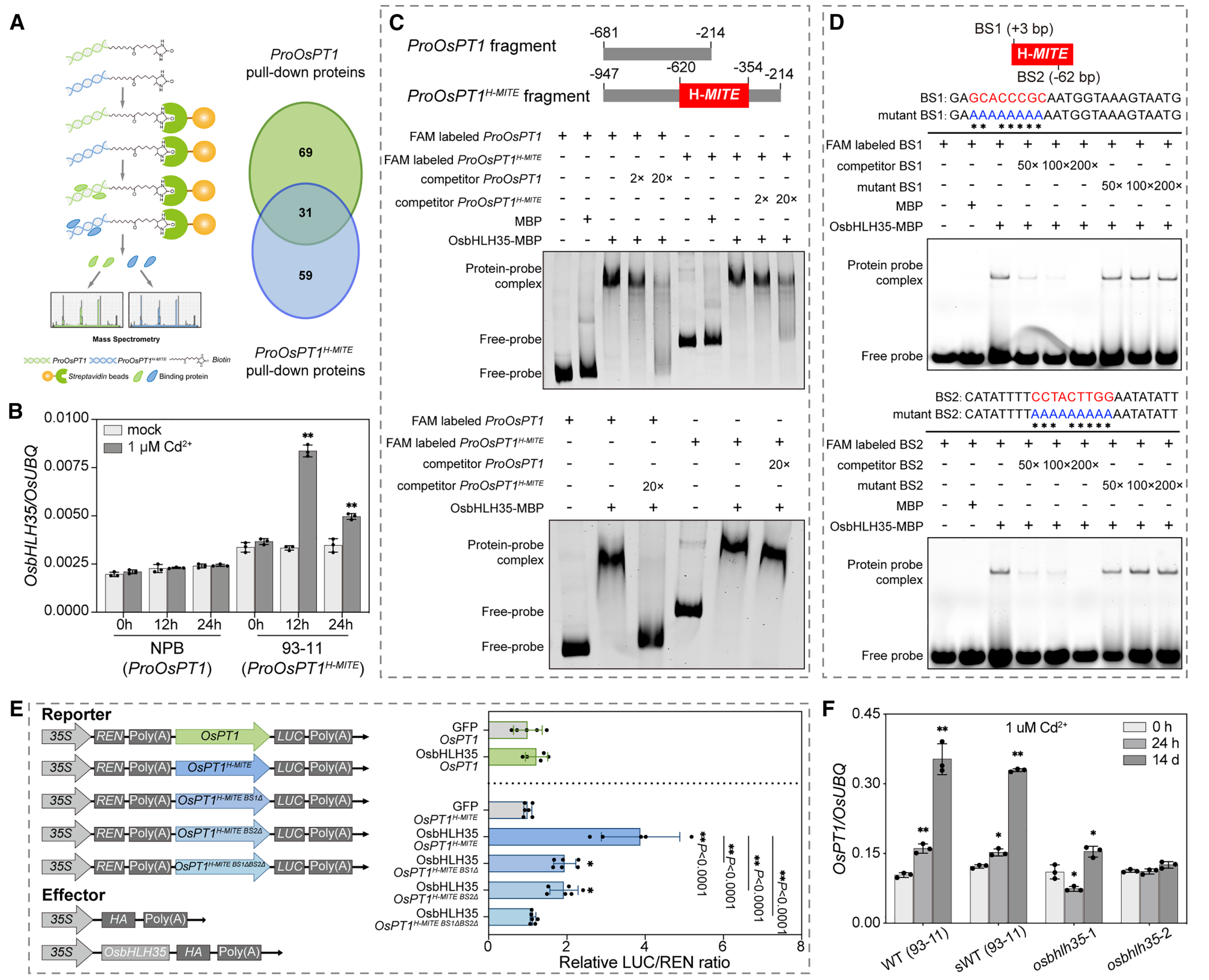

Figure 5. OsbHLH35 stably binds to two sites within *ProOsPT1$^{H\text{-}MITE}$* to activate its transcription.

(A) Identification of proteins binding to the *OsPT1* promoter with or without the H-*MITE* insertion. Left: schematic of the DNA pull-down assay. Putative binding proteins were identified by MS. Right: Venn diagram illustrating overlap between proteins that bind to *ProOsPT1* and/or *ProOsPT1$^{H\text{-}MITE}$*.

(B) Relative OsbHLH35 transcript levels in roots, as determined by RT–qPCR and normalized to *OsUBQ*. NPB, without H-*MITE* insertion in *OsPT1*; 93-11, with H-*MITE* insertion in *OsPT1*. Error bars represent SD; asterisks denote significant differences according to Student's *t*-test (**P* < 0.05, ***P* < 0.01).

(C) Electrophoretic mobility shift assay (EMSA) showing the binding of OsbHLH35 to *ProOsPT1$^{H\text{-}MITE}$* and *ProOsPT1*. FAM-labeled promoter fragments were used as probes; unlabeled competitor probes and MBP protein (negative control) served as controls. OsbHLH35 displayed stronger binding to *ProOsPT1$^{H\text{-}MITE}$* than to *ProOsPT1*.

(D) EMSA validation of OsbHLH35 binding to two specific sites (GCACCCGC and CCTACTTGG) within the H-*MITE*. Competitor and mutant probes were added at 50-, 100-, and 200-fold excess; labeled probes were used for detection.

(E) Transient dual-luciferase expression assays showing OsbHLH35-mediated transcriptional activation in rice protoplasts. Left: diagram of reporter constructs containing firefly luciferase (LUC) and *Renilla luciferase* (*REN*) reporter genes, and effector constructs (*HA* and *OsbHLH35-HA*). Right: transcriptional activation activity assays in rice protoplasts. *BS1Δ*, *BS2Δ*, and *BS1ΔBS2Δ* indicate deletions of respective BSs within the *OsPT1$^{H\text{-}MITE}$* promoter. LUC and REN activities were measured 16 h after transfection, and relative LUC/REN ratios indicate transcriptional activation. Error bars represent SD; asterisks denote significant differences according to Student's *t*-test (**P* < 0.05, ***P* < 0.01).

(F) Relative OsPT1 transcript levels in wild-type 93-11 and segregated wild type and *osbhlh35* mutants after treatment with 1 μM Cd^{2+} for 0 h, 24 h, and 14 days. Relative expression values were normalized to *OsUBQ*. Error bars represent SD; asterisks denote significant differences according to Student's *t*-test (**P* < 0.05, ***P* < 0.01).

See also Supplemental Figure 8 and Supplemental Table 9.

OsbHLH35 single and double knockout mutants, as well as lines carrying partial deletions of the H-*MITE* sequence in the 93-11 background (Supplemental Table 10). GCC was measured in mutants grown under pot cultivation in soil containing 0.85 mg/kg Cd^{2+}. The wild-type 93-11 exhibited a GCC of 1.133 ± 0.007 mg/kg (Figure 6A), whereas *ospt1* and *osbhlh35* mutants showed significantly lower GCC values of 0.272 ± 0.0008 and 0.172 mg/kg, respectively. Notably, the

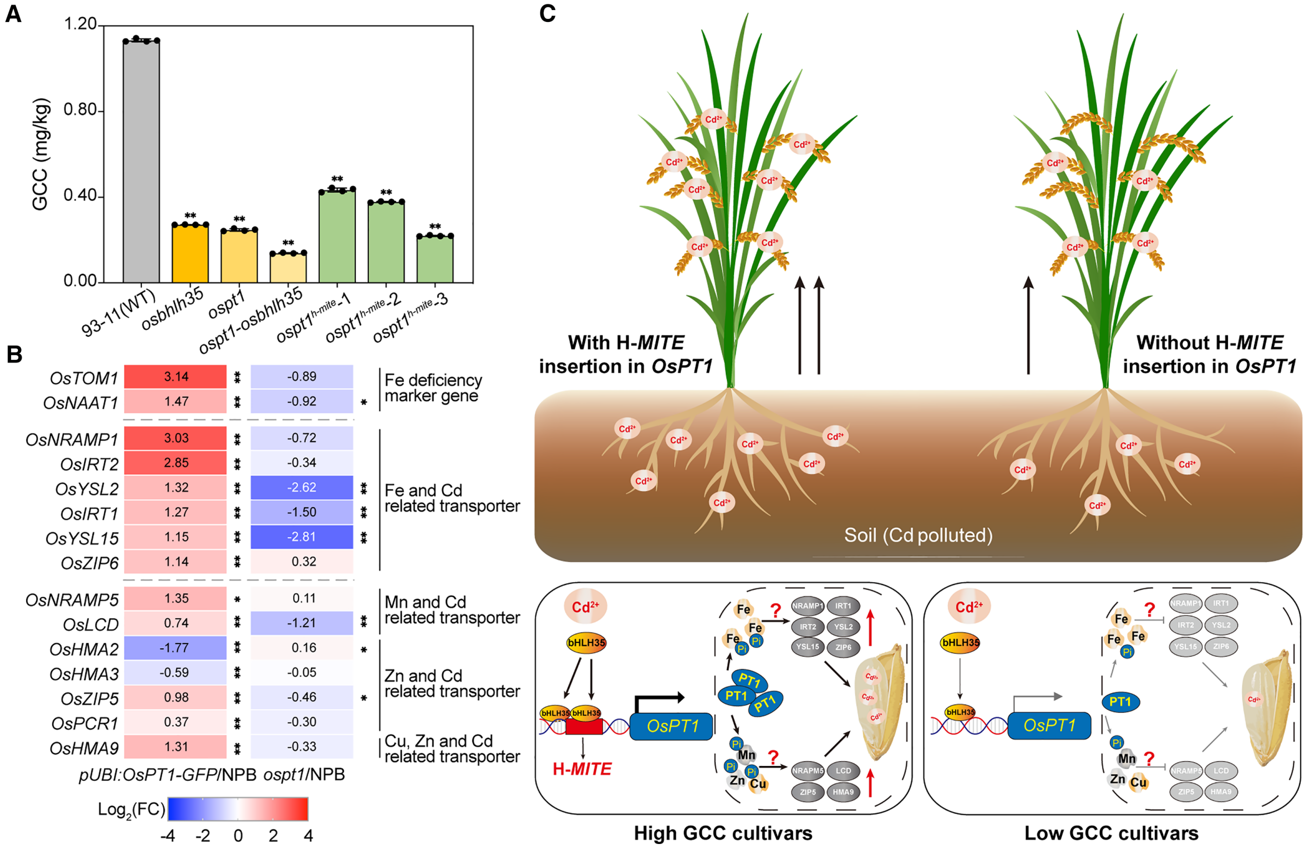

Figure 6. Knockout of *OsPT1* and *OsbHLH35* or targeted editing of H-*MITE* can significantly reduce Cd accumulation in rice grains.
(A) GCC in the 93-11 wild type, *osbhlh35* mutant, *ospt1* mutant, *ospt1 osbhlh35* double mutant, and *OsPT1*$^{H\text{-}MITE}$ targeted editing lines. Knockout or targeted editing significantly reduced GCC compared with the wild type. Error bars represent SD; asterisks denote significant differences according to Student's *t*-test (**$P < 0.01$).
(B) Heatmap showing gene expression profiles of Fe/Cd co-transporter genes in wild-type, *OsPT1* overexpression (*pUBI:OsPT1-GFP*), and *ospt1* mutant plants. Upregulated genes are shown in red and downregulated genes in blue. Error bars represent SD; asterisks denote significant differences according to Student's *t*-test (*$P < 0.05$, **$P < 0.01$).
(C) Proposed model illustrating the association between *OsPT1* and Cd accumulation in rice. In IND rice, an H-*MITE* transposon insertion in the 5′ UTR of *OsPT1* modifies the TSS and reprograms its expression pattern. The transcription factor OsbHLH35, induced by Cd stress, specifically binds to *ProOsPT1*$^{H\text{-}MITE}$ and strongly activates *OsPT1* transcription. Elevated OsPT1 expression likely disrupts ion homeostasis by influencing Pi accumulation and inducing the upregulation of Fe/Cd and Mn/Cd co-transporter genes. This process leads to Cd accumulation in rice grains (bottom left). In *japonica* rice, OsbHLH35 binds weakly to *ProOsPT1*, resulting in limited *OsPT1* activation and lower Cd accumulation in rice grains (bottom right). Dotted lines indicate hypothetical mechanisms supported by limited experimental evidence in this study. See also Supplemental Figures 9 and 10 and Supplemental Table 10.

ospt1 osbhlh35 double mutant displayed an even lower GCC of 0.139 ± 0.002 mg/kg compared with either single mutant (Figure 6A). Additionally, three mutants with partial deletions in the H-*MITE* region (*ospt1*$^{h\text{-}mite}$-1, *ospt1*$^{h\text{-}mite}$-2, and *ospt1*$^{h\text{-}mite}$-3) demonstrated GCC values of 0.434 ± 0.009, 0.378 ± 0.002, and 0.220 ± 0.002 mg/kg, respectively—all significantly reduced relative to wild-type 93-11 ($P < 0.0001$). These results indicate that CRISPR-Cas9–mediated knockout of *OsPT1* or *OsbHLH35*, as well as partial deletion of the H-*MITE* insertion, greatly reduces GCC, with reductions ranging from 61.7% to 80.6%. Furthermore, phenotypic analyses of plant height, panicle length, tiller number, effective spikes per plant, seed-setting rate, 1000-grain weight, flag leaf length, and flag leaf width revealed no significant differences between the mutant (*ospt1*, *osbhlh35*, *ospt1 osbhlh35*, and *ospt1*$^{h\text{-}mite}$) and wild type plants (Supplemental Figures 9A–9I and 10A–10D).

Taken together, these findings provide further evidence for strong associations of *OsPT1* and *OsbHLH35* with Cd accumulation. Targeted knockout of either gene in 93-11 effectively reduces GCC, whereas partial deletion of the H-*MITE* element within the 5′ UTR of *OsPT1* similarly diminishes Cd accumulation in rice grains.

Proposed mechanism of *OsPT1* function in Cd accumulation in rice

To determine whether *OsPT1* directly mediates Cd transport, a transgenic yeast strain overexpressing the rice *OsPT1* gene was generated (Yan et al., 2019). Under Cd stress conditions, *OsPT1* overexpression did not result in increased Cd content compared with the wild-type strain (Supplemental Figure 10E). This result suggests that *OsPT1* is unlikely to directly facilitate Cd transport from the extracellular environment into cells.

To further investigate why *OsPT1* overexpression is correlated with enhanced Cd accumulation in rice, we considered previous reports describing the antagonistic interaction between iron (Fe) and inorganic phosphate (Pi) in plant nutrition (Guo et al., 2022b). Elevated Pi levels have been shown to induce Fe deficiency *in planta*, which may subsequently promote Cd uptake and translocation through Fe transporters (Dekock et al., 1979; Nakanishi et al., 2006). We performed an *in vitro* assay to examine potential interactions between phosphate and Fe^{2+}; the results showed that both $(PO_4)^{3-}$ and $(H_2PO_4)^{-}$ can strongly bind Fe^{2+}, forming visible precipitates within 5 min (Supplemental Figure 10F and 10G).

Subsequently, the expression profiles of Fe deficiency marker genes were analyzed in *OsPT1* overexpression (*pUBI:OsPT1-GFP*) and *ospt1* mutant plants. The Fe deficiency markers *OsTOM1* (Nozoye et al., 2011) and *OsNAAT1* (Cheng et al., 2007) were upregulated in *OsPT1* overexpression lines but not in *ospt1* mutant plants (Figure 6B), indicating that the overexpression lines experienced Fe deficiency. In addition, we assessed expression levels of metal transporter genes associated with Cd, Fe, Zn, Mn, Mg, and Cu homeostasis via RT–qPCR. Compared with wild-type NPB, Fe- and Cd-related transporter genes—such as *OsNRAMP1*, *OsIRT2*, *OsYSL2*, *OsYSL15*, *OsIRT1*, and *OsZIP6*—were significantly upregulated ($P < 0.01$) in *OsPT1* overexpression plants (Figure 6B). Conversely, *OsYSL2*, *OsYSL15*, and *OsIRT1* were significantly downregulated ($P < 0.01$) in *ospt1* mutants. Notably, expression levels of the Mn/Cd co-transporter genes *OsNRAMP5* and *OsLCD*, the Zn/Cd-related transporter gene *OsZIP5*, and the Cu-, Zn-, and Cd-related *OsHMA9* gene were also elevated in *OsPT1* overexpression lines. Furthermore, Fe and Zn contents in shoots were measured under normal and 1 μM Cd^{2+} stress conditions. *OsPT1* overexpression plants accumulated more Fe relative to wild-type plants (Supplemental Figure 10H), whereas *ospt1* mutants showed reduced Fe accumulation (Supplemental Figure 10I). Zn levels were largely unchanged across genotypes (Supplemental Figure 10J and 10K). Collectively, these results suggest that *OsPT1* overexpression disrupts cellular ion homeostasis, leading to upregulation of Fe/Cd and Mn/Cd co-transporter genes and, consequently, enhanced Cd accumulation in rice. Although these data support links among *OsPT1* activity, phosphate–iron dynamics, and Cd uptake, further studies are needed to fully elucidate the molecular basis of these relationships.

DISCUSSION

Cd is readily absorbed by rice grown in Cd-contaminated soil and accumulates in the grains, entering the human body through the food chain and posing a serious threat to human health (Uraguchi and Fujiwara, 2013; Clemens and Ma, 2016). The development of rice varieties with low grain Cd accumulation represents an effective strategy to reduce Cd contamination. Rice cultivars with diverse genetic backgrounds exhibit substantial variation in GCC (Clemens et al., 2013). In this study, we detected substantial variation in GCC among 310 accessions from the RDP1 population grown in a Cd-contaminated field. Across rice subpopulations, IND cultivars displayed higher average GCC than *japonica* cultivars, consistent with previous reports (Jiang et al., 2008; Sun et al., 2016; Tan et al., 2020). The pronounced variation in GCC within the RDP1 population provided an excellent resource for dissecting the genetic basis of Cd accumulation in rice grains (Yan et al., 2019).

GWASs have been widely utilized to identify genomic regions linked to agronomic traits, including Cd accumulation (Zhao et al., 2011; Yang et al., 2018). In this study, we identified 29 LAGCCs, 11 of which co-localized with previously reported QTLs or candidate genes detected through conventional mapping approaches (Chen et al., 2008; Norton et al., 2009; Ueno et al., 2009; Ishikawa et al., 2010; Shimo et al., 2011; Yan et al., 2013; Yang et al., 2016). Notably, most LAGCCs identified in this study were located on chromosomes 3 and 7.

Cd is a non-essential, opportunistic metal ion transported by multiple metal transporters, including those for Fe (Nakanishi et al., 2006; Takahashi et al., 2011), Zn (Sasaki et al., 2014), and Mn (Sasaki et al., 2012). Here, we identified and characterized a major locus, LAGCC4, associated with high Cd accumulation and demonstrated that *OsPT1* within this locus is linked to GCC. *OsPT1* encodes a phosphate transporter, and other members of the OsPT family are known to mediate the transport of various elements, such as selenite (Se) by OsPT2 (Zhang et al., 2014) and arsenate (As) by OsPT4 (Ye et al., 2017). To our knowledge, this study provides the first evidence connecting a phosphate transporter with Cd accumulation in rice grains.

We found that *OsPT1* haplotypes are associated with grain Cd accumulation in rice. *OsPT1* exists as two major haplotypes (types A and B) in the RDP1 population, distinguished by a 266-bp H-*MITE* TE insertion in the 5′ UTR of *OsPT1*. Polymorphisms caused by transposon insertions are known to influence gene function (Song and Cao, 2017). For instance, a *Harbinger*-type element in maize (*Zea mays*) represses *ZmCCT9* (*CONSTANS*, *CONSTANS-LIKE*, and *TOC1 domain-containing protein -9* [CCT domain-containing protein -9]) to promot flowering under long-day conditions (Huang et al., 2018). Similarly, the *Hopscotch* transposon enhances expression of the domestication gene *Tb1*, leading to increased apical dominance in domesticated maize compared with its wild ancestor, teosinte (Studer et al., 2011). In contrast, the H-*MITE* insertion in *OsPT1* reprograms its expression under both normal and Cd stress conditions, conferring a strong Cd-dependent induction and resulting in elevated Cd accumulation in rice grains. In addition, Cd exposure induces *OsbHLH35*, a transcription factor that specifically binds to the H-*MITE*–containing *OsPT1* promoter, further enhancing *OsPT1* expression and increasing GCC.

Although *OsPT1* does not appear to directly mediate Cd transport, its overexpression leads to the upregulation of Fe/Cd transporter genes (e.g., *OsNRAMP1* and *OsIRT2*) and the Mn/Cd transporter *OsNRAMP5*, all of which are known to facilitate Cd uptake and translocation. This is likely a result of altered phosphate homeostasis disturbing the cellular balance of metal ions; both *in vitro* and *in planta* evidence indicate the formation of insoluble phosphate–Fe complexes and the induction of Fe deficiency responses under high Pi conditions (Ward et al., 2008). Indeed, the Fe deficiency marker genes *OsTOM1* and *OsNAAT1* were upregulated in *OsPT1*-overexpressing plants. Collectively, these findings suggest that *OsPT1* indirectly influences Cd

accumulation by modulating the expression of metal co-transporter genes through phosphate-mediated disruptions in ion homeostasis.

In our experiments, the maize ubiquitin promoter was used to drive *OsPT1* expression, leading to increased Cd accumulation in both roots and shoots. Although this constitutive promoter is effective for mechanistic analysis, it may induce unintended alterations in growth or mineral uptake under standard conditions. The use of targeted promoters—such as root-specific or stress-inducible elements—could confine gene activation to relevant tissues or environmental contexts, thereby minimizing side effects and enhancing applicability for crop improvement.

Previous studies have identified genes such as *CF1* and *PEZ1* that influence Cd accumulation through Fe-mediated pathways. *CF1*, an allele of the iron transporter *OsYSL2*, reduces grain Cd levels by enhancing Fe translocation from roots to shoots, thereby triggering systemic signaling that downregulates *OsNramp5*—a major Cd uptake transporter in roots (Masuda et al., 2012; Li et al., 2022). Similarly, *PEZ1* modulates Cd accumulation through competitive regulation between Fe and Cd transporters (Ishimaru et al., 2011). Both *CF1* and *PEZ1* exert indirect control over Cd accumulation, primarily through systemic regulation of iron homeostasis and Cd transporter activity. In contrast, *OsPT1* represents a distinct mechanism in which a phosphate transporter affects Cd accumulation by altering phosphate-related ion homeostasis within plant tissues. This distinction highlights different levels of biological control: *CF1* and *PEZ1* act via systemic Fe signaling and competitive regulation influencing root uptake, whereas *OsPT1* acts locally by modifying intracellular phosphate and metal ion interactions. Taken together, these complementary pathways underscore the complexity of Cd accumulation regulation.

We propose a working model to explain the elevated Cd accumulation in rice grains resulting from the H-*MITE* insertion in the *OsPT1* promoter. The H-*MITE* insertion alters *OsPT1* transcriptional regulation, leading to increased expression under Cd stress through OsbHLH35 binding. Elevated *OsPT1* expression may contribute to phosphate accumulation, which appears to induce Fe deficiency-like responses, as suggested by the upregulation of Fe deficiency marker genes. These responses may, in turn, elevate the expression of Fe/Cd and Mn/Cd co-transporter genes, thereby promoting grain Cd accumulation. Notably, IND rice cultivars carrying this insertion generally display higher GCC under Cd-contaminated field conditions. Furthermore, knockout of *OsPT1* or *OsbHLH35*, as well as partial deletion of the H-*MITE* fragment in the IND cultivar 93-11, led to reductions in GCC. Although these findings provide new insight into transporter-mediated Cd accumulation, the mechanistic connections among phosphate accumulation, Fe deficiency, and Cd uptake require further experimental confirmation.

In this study, we found that *OsPT1* disrupts cellular ion homeostasis and is positively associated with increased GCC in rice. Our findings reveal a previously uncharacterized pathway linking phosphate transport to heavy metal accumulation, highlighting the complexity of metal ion regulation in plants. However, the precise molecular mechanisms by which *OsPT1* influences Cd accumulation are not fully understood. Gene expression analyses and metal content measurements suggest that *OsPT1* overexpression alters phosphate and iron dynamics, thereby affecting the expression of Fe/Cd and Mn/Cd co-transporter genes. Nevertheless, direct experimental validation of these interactions is needed. Studies involving biochemical, genetic, and physiological approaches are warranted to elucidate the pathways and regulatory networks through which *OsPT1* modulates Cd uptake, transport, and sequestration in rice tissues. Addressing these knowledge gaps will be essential to fully understand the role of *OsPT1* and apply these insights to the breeding of rice varieties with reduced Cd content.

METHODS

Plant materials and growth conditions

A total of 310 rice accessions from the RDP1 population were cultivated at the experimental farm in Xiangyin, Hunan Province (112°51′52″E, 28°42′30″N) during both the early and late growing seasons of 2016 (Supplemental Table 1). All accessions were first germinated in seedbeds in mid-April (early season) and mid-June (late season) and subsequently transplanted into a Cd-contaminated paddy field (average soil Cd content = 0.85 mg/kg) in mid-May and mid-July, respectively (Supplemental Figure 1C). Each accession was planted in four replicates, with approximately 30 seedlings per replicate in a plot measuring 0.2× 1.0 m (width × length). At maturity, seeds were harvested separately from each plot for GCC analysis.

To minimize the effects of soil Cd heterogeneity and environmental variation, a randomized complete block design with two replicates was implemented across paddy fields with well-characterized and uniform baseline Cd levels (0.80 ± 0.05 mg/kg), as determined by inductively coupled plasma mass spectrometry (ICP–MS) before sowing. Prior to transplanting, the top 20 cm of soil in each block was thoroughly tilled and homogenized to reduce microsite variability in Cd concentration. All plots received identical basal fertilization ($N:P_2O_5:K_2O$ = 150:75:75 kg/ha) and were maintained under flooded conditions with approximately 5 cm of standing water from tillering to grain filling to ensure uniform Cd exposure. Additionally, each block was bordered by two rows of the IND cultivar ‘Xiangwanxian13’ to buffer edge effects and stabilize soil Cd diffusion. These measures collectively minimized environmental variability and improved the reliability of phenotypic measurements.

Generation of transgenic and gene-edited rice lines

The full-length coding sequence of *OsPT1* was amplified from NPB cDNA using primers cdsOsPT1-F and cdsOsPT1-R. The PCR product was first cloned into the pEASY-Blunt Zero Cloning Vector (TransGen Biotech, CB501-01) and then subcloned into the pRHVcGFP expression vector using OsPT1-F-HindIII and OsPT1-R-NotI primers. The pRHVcGFP vector utilizes the maize ubiquitin promoter to drive *OsPT1* expression. Transformation into NPB plants was performed via *Agrobacterium tumefaciens*-mediated genetic transformation.

For targeted mutagenesis of *OsPT1* and *OsbHLH35*, CRISPR-Cas9 gene editing was performed in both NPB and 93-11 backgrounds. Twenty-nucleotide target sites were selected using an online tool (http://cbi.hzau.edu.cn/CRISPR2/). Two single guide RNAs (sgRNAs) targeting distinct regions within the *OsPT1* coding sequence were designed (GACGATTC CAAGGACACCCC<u>CGG</u> and GACGATTCCAAGGACACCCC<u>CGG,</u> PAM sites underlined). The sgRNA for *OsbHLH35* targeted CAAGAGCTGC AGC-CATACGA<u>TGG</u>. To edit the highly repetitive H-*MITE* transposon in the 5′ UTR of *OsPT1*, a target site was selected near the terminal region of the H-*MITE* (AACACAATCGC-TTCTCATTA<u>GGG</u>).

To assess promoter activity, *OsPT1* promoter regions from 93-11 (containing the 266-bp H-*MITE* insertion) and NPB (lacking the 266-bp H-*MITE* insertion) were amplified using primers 1301-proOsPT1-F/R. The resulting fragments were cloned into the pCAMBIA1301 vector containing the GUS reporter gene; the constructs were introduced into NPB via *Agrobacterium tumefaciens*-mediated transformation.

Measurement of cadmium and phosphate content in rice tissues

Grain Cd concentrations were determined using an ICP–optical emission spectrometer (ICP-OES 720, Agilent Technologies, USA). Harvested rice seeds were oven-dried, cleaned, and dehusked using an electric dehusker. Dehusked grains were weighed and transferred to 100 ml digestion flasks. Each sample was treated with 5 ml of an acid mixture (4 ml HNO_3 and 1 ml $HClO_4$), pre-digested overnight at room temperature, and then completely digested at 150°C in a digestion oven. After they had cooled, the digests were diluted with Milli-Q water to a final volume of 25 ml. Cd concentrations were quantified by ICP-OES, and the same procedure was utilized for the determination of other metal elements.

Cd content in roots and shoots was analyzed using an ICP-OES (Agilent 7700 series, Agilent Technologies). Prior to analysis, tissue samples were thoroughly rinsed with deionized water (4–5 times), oven-dried at 80°C for 6 h, and digested in 65% HNO_3 using a MARS6 microwave digestion system at 180°C for 45 min. The digested solution was diluted to 15 ml with deionized water and filtered through 0.22-μm cellulose acetate membrane filters. Blank HNO_3 served as the negative control, and certified standard reference material was used as the positive control. The same procedure was used for the determination of other metal elements.

Pi content was measured as previously described (Guo et al., 2022a). Briefly, 50 mg of fresh tissue was homogenized in 0.5 ml of 5 M H_2SO_4 and 6 ml of deionized water. The homogenate was filtered into 10 ml tubes, and the pH was adjusted to 3.0. Reaction buffer (0.5 ml; 5.5 M H_2SO_4, 10 g/l ammonium molybdate, 0.5 g/l antimony potassium tartrate, 15 g/l ascorbic acid) was added to the filtrate and incubated for 30 min. Subsequently, 0.2 ml of the supernatant was used for Pi quantification by the molybdenum blue method. Absorbance was measured at 700 nm using a spectrophotometer, and Pi concentrations were calculated against a standard curve generated with known KH_2PO_4 concentrations.

GWAS and candidate gene analysis

GWASs were conducted using a high-density rice array (HDRA) containing approximately 700 000 SNPs across RDP1 accessions (available at http://www.ricediversity.org/data/index.cfm/). Genotype data were processed and filtered using PLINK version 1.9. The kinship matrix was calculated with EMMAX (Kang et al., 2010), and population structure (Q matrix) was inferred using fastStructure (Raj et al., 2014). The GWASs were performed using a mixed linear model implemented in EMMAX, which considers both population structure and kinship to minimize confounding effects. The significance threshold for association was established at $p < 1 \times 10^{-4}$. Manhattan plots were generated using the R package CMplot. LD values were calculated in TASSEL (Bradbury et al., 2007) (v5.2.48), and LD heatmaps were visualized using the Perl SVG module. For candidate gene identification, genomic intervals of 200 kb containing at least two significantly associated SNPs were defined, based on the average LD decay distance in rice. Genes within these regions were annotated and further analyzed for potential involvement in the trait of interest.

Hydroponic experiments

Hydroponic experiments were conducted using Yoshida nutrient solution as described by the International Rice Research Institute. Full-strength Yoshida solution contained the following components: 1.44 mM NH_4NO_3, 0.26 mM $NaH_2PO_4 \cdot 2H_2O$, 0.41 mM K_2SO_4, 0.8 mM $CaCl_2$, 1.31 mM $MgSO_4 \cdot 7H_2O$, 0.09 mM $FeSO_4$, 7.58 μM $MnCl_2 \cdot 4H_2O$, 0.06 μM $(NH_4)_6Mo_7O_{24} \cdot 4H_2O$, 15 μM H_3BO_3, 0.12 μM $ZnSO_4 \cdot 7H_2O$, 0.12 μM $CuSO_4 \cdot 5H_2O$, and 4.76 μM citric acid monohydrate. The pH of the solution was adjusted to 5.7.

Rice seeds were surface-sterilized with 10% sodium hypochlorite, thoroughly rinsed with sterile water, and incubated at 30°C for 48 h to promote germination. Germinated seeds were transferred to 96-well plates with the bottoms removed and cultured in Yoshida solution supplemented with either 1 or 5 μM Cd^{2+}. The nutrient solution was renewed every 2 days.

All rice lines, including NPB, 93-11, and transgenic lines, were grown in a controlled growth chamber under a 14 h light/10 h dark photoperiod at 28°C/26°C (day/night) with approximately 60% relative humidity. Seedling and hydroponic phenotypes were assessed under both Cd-treated and untreated conditions as described.

Sequencing and sequence analysis of Cd content-associated genes

Sixteen rice accessions with low grain Cd accumulation and 10 with high grain Cd accumulation were selected for candidate gene sequencing. Genomic DNA was extracted from seedling tissues using the cetyltrimethylammonium bromide (CTAB) method. Gene-specific primers (listed in Supplemental Table 11) were designed based on the MSU v7.0 rice genome reference (http://rice.plantbiology.msu.edu/). PCR amplification was performed for five transporter-encoding genes in all 26 accessions. Amplified products were subjected to Sanger sequencing (TsingKe BioTech, Beijing, China). Sequence assembly and alignment were conducted using DNASTAR and MEGA7, following default parameters unless otherwise indicated. All sequence variants were confirmed by manual inspection. Detailed sample information is provided in Supplemental Table 5.

Identification of polymorphic TEs

Identification of polymorphic TEs from high-throughput short-read sequencing data was performed as previously described (Kang et al., 2016). Briefly, paired-end reads were aligned to the reference genome using the Burrows-Wheeler Aligner (v0.7.17). Discordant read pairs and split reads indicative of TE insertions or excisions were detected. Candidate polymorphic TEs were defined based on reads spanning putative insertion sites and stringently filtered to minimize false positives.

5′ RACE

The TSSs of *OsPT1$^{H\text{-}MITE}$* and *OsPT1* were determined using the HiScript-TS 5′/3′ RACE Kit (Vazyme, RA101), in accordance with the manufacturer's instructions. Nested PCR was performed with a 5′ coding sequence primer and gene-specific primers listed in Supplemental Table 11. The resulting 5′ RACE PCR products were cloned into the pEASY-Blunt Zero Cloning Vector (TransGen Biotech, CB501-01) to precisely map the TSSs. Clones were sequenced by TsingKe BioTech.

Histochemical staining of GUS activity

Histochemical staining of GUS activity was performed on *ProOsPT1$^{H\text{-}MITE}$::GUS* and *ProOsPT1::GUS* transgenic plants. Briefly, plant tissues were incubated overnight at 37°C in staining buffer containing 0.5 mM X-Gluc. After staining, plant tissues were destained in 75% ethanol for 12 h; ethanol was renewed every 2 h. Samples were then embedded in 2.5% (w/w) agar and sectioned into 35-μm slices using a Leica VT 1000 S vibratome. Thin sections were observed and imaged with a Leica DM6000M microscope.

Biotin–streptavidin DNA pull-down assay

To identify proteins binding to the *OsPT1* promoter, biotin–streptavidin pull-down assays were performed. Briefly, promoter fragments of 468 bp (*ProOsPT1*) and 734 bp (*ProOsPT1$^{H\text{-}MITE}$*) were PCR-amplified with biotin-labeled primers (biotin-PT1-F/PT1-R) and purified using a

PCR purification kit. Total protein was extracted from rice leaves using extraction buffer (100 mM Tris-HCl [pH 7.5], 150 mM NaCl, 1 mM ethylenediaminetetraacetic acid [EDTA; pH 8.0], 0.5% Nonidet P-40, and 1 mM dithiothreitol).

Streptavidin magnetic beads (100 μl) were washed twice with phosphate-buffered saline (PBS) and incubated with biotinylated promoter fragments in PBS at 4°C for 2 h with gentle rotation to immobilize the DNA. The DNA-bound beads were then incubated overnight at 4°C with total protein extracts under continuous agitation. After incubation, beads were washed three times with wash buffer (PBS containing 0.1% Tween 20), and bound proteins were eluted using elution buffer at 95°C for 5 min. Eluted proteins were subsequently analyzed by MS.

Protein mass spectrometry (MS) analysis

Proteins pulled down by biotinylated *ProOsPT1* (468 bp) and *ProOsPT1*$^{H\text{-}MITE}$ (734 bp) fragments were subjected to MS analysis. Purified proteins were digested overnight with sequencing-grade trypsin at 37°C. The resulting peptides were analyzed by LC–MS/MS using a Q Exactive mass spectrometer coupled with an Easy-nLC1000 system (Thermo Fisher Scientific). The raw MS data were searched against the rice protein databases NCBI_oryza_sativa_2112565_20210419 and uniprot_Oryza_sativa_ 194 927_20210412 using MASCOT and Proteome Discoverer 1.4 software. Search parameters allowed up to two missed cleavages, with a false discovery rate threshold of 1%. Peptide fragments were mapped to the rice reference proteome (MSUv7.0). Proteins specifically enriched in the *ProOsPT1*$^{H\text{-}MITE}$ pull-down but absent in the *ProOsPT1* pull-down were selected for further analysis. All identified proteins are listed in Supplemental Table 9.

Electrophoresis mobility shift assay (EMSA)

MBP-tagged OsbHLH35, OsMADS79, and OsNAC46 proteins were heterologously expressed in *Escherichia coli* BL21 (DE3) (TransGen Biotech, CD801) and purified using amylose resin (New England Biolabs, E8021V), in accordance with the manufacturer's instructions. Protein concentrations were determined by NanoDrop, and purified proteins were stored at −80°C. The *ProOsPT1* (468 bp) and *ProOsPT1*$^{H\text{-}MITE}$ (734 bp) fragments were PCR-amplified and labeled with 5′-FAM on the sense strand.

EMSA reactions were performed as previously described with minor modifications. Briefly, 2 μl of binding buffer (10 mM Tris-HCl [pH 7.5], 50 mM KCl, 3.5 mM dithiothreitol, 0.25% Tween 20, 5% glycerol, 5 mM $MgCl_2$, and 50 mM EDTA) was mixed with labeled probe, purified protein, and 1 mg poly(dI-dC) as a nonspecific competitor. Reactions were incubated at room temperature for 20 min in the presence or absence of unlabeled competitor probes. Samples were resolved on 4% native polyacrylamide gels in 0.5× Tris/borate/EDTA buffer at 4°C. The MBP protein alone served as a negative control. Fluorescent signals were detected using a Typhoon 9410 scanner (GE Healthcare).

Transcriptional activity assay in rice protoplasts

The transcriptional activity assay was performed as previously described. Rice protoplast preparation and transfection were performed following established protocols (Fan et al., 2018). In brief, promoter sequences of *OsPT1* (468 bp) and *OsPT1*$^{H\text{-}MITE}$ (734 bp) were PCR-amplified from genomic DNA and cloned into the pGreenII 0800-LUC vector, which contains a *Renilla luciferase* (*REN*) gene driven by the CaMV 35S promoter as an internal control. The coding sequence of *OsbHLH35* was cloned into the pYBA1143 vector under the control of the 35S promoter to serve as the effector.

For each transfection, 2 μg of plasmid DNA was used for each reporter and effector construct. Protoplasts were transfected using the polyethylene glycol (PEG)–mediated method and incubated for 12 h at 28°C. Luciferase activities were measured using the Dual-Luciferase Reporter Assay System (Promega), in accordance with the manufacturer's instructions. Relative luciferase activity was calculated by normalizing LUC activity to REN activity.

Yeast one-hybrid (Y1H) assay

The yeast one-hybrid assay was performed using the Y1HGold yeast strain and the pAbAi reporter system (Coolaber, Beijing, China) to examine protein–DNA interactions. Promoter sequences of *OsPT1* (468 bp) and *OsPT1*$^{H\text{-}MITE}$ (734 bp) were cloned into the pAbAi vector upstream of the Aureobasidin A (*AbA*) resistance gene. Recombinant pAbAi plasmids were linearized and integrated into the Y1HGold genome via transformation according to the manufacturer's protocol.

The coding sequence of *OsbHLH35* was cloned into the pGADT7-AD vector to generate activation domain fusion constructs. These prey plasmids were introduced into the bait-containing Y1HGold strains using the lithium acetate transformation method.

Transformants were selected on synthetic dropout medium lacking leucine (synthetic dropout/−Leu) to maintain the pGADT7-AD plasmid. Positive interactions were identified by growth on synthetic dropout/−Leu medium supplemented with an optimized concentration of AbA, as determined by preliminary minimal inhibitory concentration assays. Plates were incubated at 30°C for 3–5 days before assessing yeast growth.

Quantification and statistical analysis

Multiple comparisons were performed using GraphPad Prism version 9.00 for Mac OS with one-way analysis of variance (ANOVA) followed by Dunnett's multiple comparisons test. Pairwise comparisons were conducted using Student's *t*-test in Microsoft Excel. Broad-sense heritability was calculated as $H^2 = \sigma g^2 / (\sigma g^2 + \sigma e^2/n)$, where σg^2 represents genotypic variance, σe^2 represents environmental variance, and *n* is the number of replications.

Accession numbers

The gene sequences have been deposited in NCBI GenBank under accession numbers OM681530–OM681558 (Supplemental Table 12).

FUNDING

This work was supported by the National Natural Science Foundation of China (grants 32261143468 and U24A20405) and the High Technology Industry S&T Innovation Leading Project of Hunan Province (2020NK2001).

ACKNOWLEDGMENTS

We thank Dr. Zhilong Wang for his contributions at the early stages of project implementation. The State Key Laboratory for Biology of Plant Diseases and Insect Pests and the Hunan Provincial Key Laboratory of Crop Germplasm Innovation and Utilization contributed equally to this study. No conflict of interest is declared.

AUTHOR CONTRIBUTIONS

H.K., J.L., and L.B. designed the research. S.P., J.L., S.J., Y. Xu, and Y.P. conducted the research. H.A., Y. Xiao, K.Y. J.W., J.Z., and B.L contributed to the experiments. S.P., H.K., and J.L. performed the GWASs. S.P., D.W., S.J., Y.D., Z.X., F.H., Z.L., and H.A. contributed to field management. S.P. and H.K. analyzed the data. H.K. developed the database and bioinformatics tools. S.P., H.K., and G.-L.W. analyzed the data and wrote the manuscript.

SUPPLEMENTAL INFORMATION

Supplemental information is available at *Plant Communications Online*.

Received: September 5, 2025
Revised: September 26, 2025
Accepted: October 14, 2025
Published: October 15, 2025

REFERENCES

Aoshima, K. (2017). Itai-itai disease: Lessons from the investigations of environmental epidemiology conducted in the 1970's, with special reference to the studies of the Toyama Institute of Health. Nihon Eiseigaku Zasshi **72**:149–158. https://doi.org/10.1265/jjh.72.149.

Bradbury, P.J., Zhang, Z., Kroon, D.E., Casstevens, T.M., Ramdoss, Y., and Buckler, E.S. (2007). TASSEL: software for association mapping of complex traits in diverse samples. Bioinformatics **23**:2633–2635. https://doi.org/10.1093/bioinformatics/btm308.

Chen, W.R., Feng, Y., and Chao, Y.E. (2008). Genomic analysis and expression pattern of OsZIP1, OsZIP3, and OsZIP4 in two rice (Oryza sativa L.) genotypes with different zinc efficiency. Russ. J. Plant Physiol. **55**:400–409. https://doi.org/10.1134/S1021443708030175.

Cheng, L., Wang, F., Shou, H., Huang, F., Zheng, L., He, F., Li, J., Zhao, F.J., Ueno, D., Ma, J.F., and Wu, P. (2007). Mutation in nicotianamine aminotransferase stimulated the Fe(II) acquisition system and led to iron accumulation in rice. Plant Physiol. **145**:1647–1657. https://doi.org/10.1104/pp.107.107912.

Chow, C.N., Lee, T.Y., Hung, Y.C., Li, G.Z., Tseng, K.C., Liu, Y.H., Kuo, P.L., Zheng, H.Q., and Chang, W.C. (2019). PlantPAN3.0: a new and updated resource for reconstructing transcriptional regulatory networks from ChIP-seq experiments in plants. Nucleic Acids Res. **47**:D1155–D1163. https://doi.org/10.1093/nar/gky1081.

Clemens, S., and Ma, J.F. (2016). Toxic Heavy Metal and Metalloid Accumulation in Crop Plants and Foods. Annu. Rev. Plant Biol. **67**:489–512. https://doi.org/10.1146/annurev-arplant-043015-112301.

Clemens, S., Aarts, M.G.M., Thomine, S., and Verbruggen, N. (2013). Plant science: the key to preventing slow cadmium poisoning. Trends Plant Sci. **18**:92–99. https://doi.org/10.1016/j.tplants.2012.08.003.

Dekock, P.C., Hall, A., and Inkson, R.H.E. (1979). Active Iron in Plant Leaves. Ann. Bot. **43**:737–740. https://doi.org/10.1093/oxfordjournals.aob.a085687.

Eizenga, G.C., Ali, M.L., Bryant, R.J., Yeater, K.M., McClung, A.M., and McCouch, S.R. (2014). Registration of the Rice Diversity Panel 1 for Genomewide Association Studies. J. Plant Registrations **8**:109–116. https://doi.org/10.3198/jpr2013.03.0013crmp.

Fan, J., Bai, P., Ning, Y., Wang, J., Shi, X., Xiong, Y., Zhang, K., He, F., Zhang, C., Wang, R., et al. (2018). The Monocot-Specific Receptor-like Kinase SDS2 Controls Cell Death and Immunity in Rice. Cell Host Microbe **23**:498–510.e5. https://doi.org/10.1016/j.chom.2018.03.003.

Farooq, M.A., Detterbeck, A., Clemens, S., and Dietz, K.J. (2016). Silicon-induced reversibility of cadmium toxicity in rice. J. Exp. Bot. **67**:3573–3585. https://doi.org/10.1093/jxb/erw175.

Gao, Y., Duan, Z., Zhang, L., Sun, D., and Li, X. (2022). The Status and Research Progress of Cadmium Pollution in Rice- (Oryza sativa L.) and Wheat- (Triticum aestivum L.) Cropping Systems in China: A Critical Review. Toxics **10**:10120794. https://doi.org/10.3390/toxics10120794.

Guo, M., Zhang, Y., Jia, X., Wang, X., Zhang, Y., Liu, J., Yang, Q., Ruan, W., and Yi, K. (2022a). Alternative splicing of REGULATOR OF LEAF INCLINATION 1 modulates phosphate starvation signaling and growth in plants. Plant Cell **34**:3319–3338. https://doi.org/10.1093/plcell/koac161.

Guo, M., Ruan, W., Zhang, Y., Zhang, Y., Wang, X., Guo, Z., Wang, L., Zhou, T., Paz-Ares, J., and Yi, K. (2022b). A reciprocal inhibitory module for Pi and iron signaling. Mol. Plant **15**:138–150. https://doi.org/10.1016/j.molp.2021.09.011.

Honma, T., Ohba, H., Kaneko-Kadokura, A., Makino, T., Nakamura, K., and Katou, H. (2016). Optimal Soil Eh, pH, and Water Management for Simultaneously Minimizing Arsenic and Cadmium Concentrations in Rice Grains. Environ. Sci. Technol. **50**:4178–4185. https://doi.org/10.1021/acs.est.5b05424.

Hu, R., Limmer, M.A., and Seyfferth, A.L. (2024). How manganese affects rice cadmium uptake and translocation in vegetative and mature plants. Plant Soil **504**:941–954. https://doi.org/10.1007/s11104-024-06669-9.

Huang, C., Sun, H., Xu, D., Chen, Q., Liang, Y., Wang, X., Xu, G., Tian, J., Wang, C., Li, D., et al. (2018). ZmCCT9 enhances maize adaptation to higher latitudes. Proc. Natl. Acad. Sci. USA **115**:E334–E341. https://doi.org/10.1073/pnas.1718058115.

Huang, N., Wang, B., Liu, S., Wang, K., Wang, R., Liu, F., and Chen, C. (2025). Cadmium exposure in infants and children: toxicity, health effects, dietary risk assessment and mitigation strategies. Crit. Rev. Food Sci. Nutr. **65**:5085–5107. https://doi.org/10.1080/10408398.2024.2403036.

Huang, S., Yamaji, N., and Ma, J.F. (2024b). Metal Transport Systems in Plants. Annu. Rev. Plant Biol. **75**:1–25. https://doi.org/10.1146/annurev-arplant-062923-021424.

Huang, X., Wei, X., Sang, T., Zhao, Q., Feng, Q., Zhao, Y., Li, C., Zhu, C., Lu, T., Zhang, Z., et al. (2010). Genome-wide association studies of 14 agronomic traits in rice landraces. Nat. Genet. **42**:961–967. https://doi.org/10.1038/ng.695.

Ishikawa, S., Abe, T., Kuramata, M., Yamaguchi, M., Ando, T., Yamamoto, T., and Yano, M. (2010). A major quantitative trait locus for increasing cadmium-specific concentration in rice grain is located on the short arm of chromosome 7. J. Exp. Bot. **61**:923–934. https://doi.org/10.1093/jxb/erp360.

Ishikawa, S., Ishimaru, Y., Igura, M., Kuramata, M., Abe, T., Senoura, T., Hase, Y., Arao, T., Nishizawa, N.K., and Nakanishi, H. (2012). Ion-beam irradiation, gene identification, and marker-assisted breeding in the development of low-cadmium rice. Proc. Natl. Acad. Sci. USA **109**:19166–19171. https://doi.org/10.1073/pnas.1211132109.

Ishimaru, Y., Bashir, K., Nakanishi, H., and Nishizawa, N.K. (2011). The role of rice phenolics efflux transporter in solubilizing apoplasmic iron. Plant Signal. Behav. **6**:1624–1626. https://doi.org/10.4161/psb.6.10.17694.

Jia, L., Mao, Y., Ji, Q., Dersh, D., Yewdell, J.W., and Qian, S.B. (2020). Decoding mRNA translatability and stability from the 5' UTR. Nat. Struct. Mol. Biol. **27**:814–821. https://doi.org/10.1038/s41594-020-0465-x.

Jiang, S.L., Wu, J.G., Thang, N.B., Feng, Y., Yang, X.E., and Shi, C.H. (2008). Genotypic variation of mineral elements contents in rice (Oryza sativa L.). Eur. Food Res. Technol. **228**:115–122. https://doi.org/10.1007/s00217-008-0914-y.

Kang, H., Zhu, D., Lin, R., Opiyo, S.O., Jiang, N., Shiu, S.H., and Wang, G.L. (2016). A novel method for identifying polymorphic transposable elements via scanning of high-throughput short reads. DNA Res. **23**:241–251. https://doi.org/10.1093/dnares/dsw011.

Kang, H.M., Sul, J.H., Service, S.K., Zaitlen, N.A., Kong, S.Y., Freimer, N.B., Sabatti, C., and Eskin, E. (2010). Variance component model to account for sample structure in genome-wide association studies. Nat. Genet. **42**:348–354. https://doi.org/10.1038/ng.548.

Kumagai, S., Suzuki, T., Tezuka, K., Satoh-Nagasawa, N., Takahashi, H., Sakurai, K., Watanabe, A., Fujimura, T., and Akagi, H. (2014). Functional analysis of the C-terminal region of the vacuolar cadmium-transporting rice OsHMA3. FEBS Lett. **588**:789–794. https://doi.org/10.1016/j.febslet.2014.01.037.

Li, L., Mao, D., Sun, L., Wang, R., Tan, L., Zhu, Y., Huang, H., Peng, C., Zhao, Y., Wang, J., et al. (2022). CF1 reduces grain-cadmium levels in rice (Oryza sativa). Plant J. **110**:1305–1318. https://doi.org/10.1111/tpj.15736.

Ma, J.F., Shen, R.F., and Shao, J.F. (2021). Transport of cadmium from soil to grain in cereal crops: A review. Pedosphere **31**:3–10. https://doi.org/10.1016/s1002-0160(20)60015-7.

Masuda, H., Ishimaru, Y., Aung, M.S., Kobayashi, T., Kakei, Y., Takahashi, M., Higuchi, K., Nakanishi, H., and Nishizawa, N.K. (2012). Iron biofortification in rice by the introduction of multiple genes involved in iron nutrition. Sci. Rep. **2**:543. https://doi.org/10.1038/srep00543.

Mather, K.A., Caicedo, A.L., Polato, N.R., Olsen, K.M., McCouch, S., and Purugganan, M.D. (2007). The extent of linkage disequilibrium in rice (Oryza sativa L.). Genetics **177**:2223–2232. https://doi.org/10.1534/genetics.107.079616.

Nakanishi, H., Ogawa, I., Ishimaru, Y., Mori, S., and Nishizawa, N.K. (2006). Iron deficiency enhances cadmium uptake and translocation mediated by the Fe2+ transporters OsIRT1 and OsIRT2 in rice. Soil Sci. Plant Nutr. **52**:464–469. https://doi.org/10.1111/j.1747-0765.2006.00055.x.

Norton, G.J., Deacon, C.M., Xiong, L., Huang, S., Meharg, A.A., and Price, A.H. (2009). Genetic mapping of the rice ionome in leaves and grain: identification of QTLs for 17 elements including arsenic, cadmium, iron and selenium. Plant Soil **329**:139–153. https://doi.org/10.1007/s11104-009-0141-8.

Nozoye, T., Nagasaka, S., Kobayashi, T., Takahashi, M., Sato, Y., Sato, Y., Uozumi, N., Nakanishi, H., and Nishizawa, N.K. (2011). Phytosiderophore efflux transporters are crucial for iron acquisition in graminaceous plants. J. Biol. Chem. **286**:5446–5454. https://doi.org/10.1074/jbc.M110.180026.

Raj, A., Stephens, M., and Pritchard, J.K. (2014). fastSTRUCTURE: variational inference of population structure in large SNP data sets. Genetics **197**:573–589. https://doi.org/10.1534/genetics.114.164350.

Sasaki, A., Yamaji, N., and Ma, J.F. (2014). Overexpression of OsHMA3 enhances Cd tolerance and expression of Zn transporter genes in rice. J. Exp. Bot. **65**:6013–6021. https://doi.org/10.1093/jxb/eru340.

Sasaki, A., Yamaji, N., Yokosho, K., and Ma, J.F. (2012). Nramp5 is a major transporter responsible for manganese and cadmium uptake in rice. Plant Cell **24**:2155–2167. https://doi.org/10.1105/tpc.112.096925.

Seo, H.M., Jung, Y., Song, S., Kim, Y., Kwon, T., Kim, D.H., Jeung, S.J., Yi, Y.B., Yi, G., Nam, M.H., and Nam, J. (2008). Increased expression of OsPT1, a high-affinity phosphate transporter, enhances phosphate acquisition in rice. Biotechnol. Lett. **30**:1833–1838. https://doi.org/10.1007/s10529-008-9757-7.

Shimo, H., Ishimaru, Y., An, G., Yamakawa, T., Nakanishi, H., and Nishizawa, N.K. (2011). Low cadmium (LCD), a novel gene related to cadmium tolerance and accumulation in rice. J. Exp. Bot. **62**:5727–5734. https://doi.org/10.1093/jxb/err300.

Song, W.E., Chen, S.B., Liu, J.F., Chen, L., Song, N.N., Li, N., and Liu, B. (2015). Variation of Cd concentration in various rice cultivars and derivation of cadmium toxicity thresholds for paddy soil by species-sensitivity distribution. J. Integr. Agric. **14**:1845–1854. https://doi.org/10.1016/S2095-3119(14)60926-6.

Song, X., and Cao, X. (2017). Transposon-mediated epigenetic regulation contributes to phenotypic diversity and environmental adaptation in rice. Curr. Opin. Plant Biol. **36**:111–118. https://doi.org/10.1016/j.pbi.2017.02.004.

Studer, A., Zhao, Q., Ross-Ibarra, J., and Doebley, J. (2011). Identification of a functional transposon insertion in the maize domestication gene tb1. Nat. Genet. **43**:1160–1163. https://doi.org/10.1038/ng.942.

Sun, L., Tan, Y., and Chen, C. (2023). The road toward Cd-safe rice: From mass selection to marker-assisted selection and genetic manipulation. The Crop Journal **11**:1059–1071. https://doi.org/10.1016/j.cj.2023.01.007.

Sun, L., Xu, X., Jiang, Y., Zhu, Q., Yang, F., Zhou, J., Yang, Y., Huang, Z., Li, A., Chen, L., et al. (2016). Genetic Diversity, Rather than Cultivar Type, Determines Relative Grain Cd Accumulation in Hybrid Rice. Front. Plant Sci. **7**:1407. https://doi.org/10.3389/fpls.2016.01407.

Sun, S., Gu, M., Cao, Y., Huang, X., Zhang, X., Ai, P., Zhao, J., Fan, X., and Xu, G. (2012). A constitutive expressed phosphate transporter, OsPht1;1, modulates phosphate uptake and translocation in phosphate-replete rice. Plant Physiol. **159**:1571–1581. https://doi.org/10.1104/pp.112.196345.

Takahashi, R., Ishimaru, Y., Senoura, T., Shimo, H., Ishikawa, S., Arao, T., Nakanishi, H., and Nishizawa, N.K. (2011). The OsNRAMP1 iron transporter is involved in Cd accumulation in rice. J. Exp. Bot. **62**:4843–4850. https://doi.org/10.1093/jxb/err136.

Tan, Y., Sun, L., Song, Q., Mao, D., Zhou, J., Jiang, Y., Wang, J., Fan, T., Zhu, Q., Huang, D., et al. (2020). Genetic architecture of subspecies divergence in trace mineral accumulation and elemental correlations in the rice grain. Theor. Appl. Genet. **133**:529–545. https://doi.org/10.1007/s00122-019-03485-z.

Ueno, D., Kono, I., Yokosho, K., Ando, T., Yano, M., and Ma, J.F. (2009). A major quantitative trait locus controlling cadmium translocation in rice (Oryza sativa). New Phytol. **182**:644–653. https://doi.org/10.1111/j.1469-8137.2009.02784.x.

Ueno, D., Yamaji, N., Kono, I., Huang, C.F., Ando, T., Yano, M., and Ma, J.F. (2010). Gene limiting cadmium accumulation in rice. Proc. Natl. Acad. Sci. USA **107**:16500–16505. https://doi.org/10.1073/pnas.1005396107.

Uraguchi, S., and Fujiwara, T. (2013). Rice breaks ground for cadmium-free cereals. Curr. Opin. Plant Biol. **16**:328–334. https://doi.org/10.1016/j.pbi.2013.03.012.

Uraguchi, S., Kamiya, T., Sakamoto, T., Kasai, K., Sato, Y., Nagamura, Y., Yoshida, A., Kyozuka, J., Ishikawa, S., and Fujiwara, T. (2011). Low-affinity cation transporter (OsLCT1) regulates cadmium transport into rice grains. Proc. Natl. Acad. Sci. USA **108**:20959–20964. https://doi.org/10.1073/pnas.1116531109.

Wang, W., Mauleon, R., Hu, Z., Chebotarov, D., Tai, S., Wu, Z., Li, M., Zheng, T., Fuentes, R.R., Zhang, F., et al. (2018). Genomic variation in 3,010 diverse accessions of Asian cultivated rice. Nature **557**:43–49. https://doi.org/10.1038/s41586-018-0063-9.

Ward, J.T., Lahner, B., Yakubova, E., Salt, D.E., and Raghothama, K.G. (2008). The effect of iron on the primary root elongation of Arabidopsis during phosphate deficiency. Plant Physiol. **147**:1181–1191. https://doi.org/10.1104/pp.108.118562.

Yamaji, N., and Ma, J.F. (2017). Node-controlled allocation of mineral elements in Poaceae. Curr. Opin. Plant Biol. **39**:18–24. https://doi.org/10.1016/j.pbi.2017.05.002.

Yan, H., Xu, W., Xie, J., Gao, Y., Wu, L., Sun, L., Feng, L., Chen, X., Zhang, T., Dai, C., et al. (2019). Variation of a major facilitator superfamily gene contributes to differential cadmium accumulation between rice subspecies. Nat. Commun. **10**:2562. https://doi.org/10.1038/s41467-019-10544-y.

Yan, Y.F., Lestari, P., Lee, K.J., Kim, M.Y., Lee, S.H., and Lee, B.W. (2013). Identification of quantitative trait loci for cadmium accumulation and distribution in rice (Oryza sativa). Genome **56**:227–232. https://doi.org/10.1139/gen-2012-0106.

Yang, J., Gao, M.X., Hu, H., Ding, X.M., Lin, H.W., Wang, L., Xu, J.M., Mao, C.Z., Zhao, F.J., and Wu, Z.C. (2016). OsCLT1, a CRT-like transporter 1, is required for glutathione homeostasis and arsenic tolerance in rice. New Phytol. **211**:658–670. https://doi.org/10.1111/nph.13908.

Yang, M., Lu, K., Zhao, F.J., Xie, W., Ramakrishna, P., Wang, G., Du, Q., Liang, L., Sun, C., Zhao, H., et al. (2018). Genome-Wide Association Studies Reveal the Genetic Basis of Ionomic Variation in Rice. Plant Cell **30**:2720–2740. https://doi.org/10.1105/tpc.18.00375.

Ye, Y., Li, P., Xu, T., Zeng, L., Cheng, D., Yang, M., Luo, J., and Lian, X. (2017). OsPT4 Contributes to Arsenate Uptake and Transport in Rice. Front. Plant Sci. **8**:2197. https://doi.org/10.3389/fpls.2017.02197.

Yu, E., Wang, W., Yamaji, N., Fukuoka, S., Che, J., Ueno, D., Ando, T., Deng, F., Hori, K., Yano, M., et al. (2022). Duplication of a manganese/cadmium transporter gene reduces cadmium accumulation in rice grain. Nat. Food **3**:597–607. https://doi.org/10.1038/s43016-022-00569-w.

Zhang, L., Hu, B., Li, W., Che, R., Deng, K., Li, H., Yu, F., Ling, H., Li, Y., and Chu, C. (2014). OsPT2, a phosphate transporter, is involved in the active uptake of selenite in rice. New Phytol. **201**:1183–1191. https://doi.org/10.1111/nph.12596.

Zhao, D., Wang, P., and Zhao, F.J. (2024). Toxic Metals and Metalloids in Food: Current Status, Health Risks, and Mitigation Strategies. Curr. Environ. Health Rep. **11**:468–483. https://doi.org/10.1007/s40572-024-00462-7.

Zhao, F.J., Tang, Z., Song, J.J., Huang, X.Y., and Wang, P. (2022). Toxic metals and metalloids: Uptake, transport, detoxification, phytoremediation, and crop improvement for safer food. Mol. Plant **15**:27–44. https://doi.org/10.1016/j.molp.2021.09.016.

Zhao, K., Tung, C.W., Eizenga, G.C., Wright, M.H., Ali, M.L., Price, A. H., Norton, G.J., Islam, M.R., Reynolds, A., Mezey, J., et al. (2011). Genome-wide association mapping reveals a rich genetic architecture of complex traits in Oryza sativa. Nat. Commun. **2**:467. https://doi.org/10.1038/ncomms1467.

Zhong, S., Li, X., Fang, L., Bai, J., Gao, R., Huang, Y., Huang, Y., Liu, Y., Liu, C., Yin, H., et al. (2024). Multifunctional Roles of Zinc in Cadmium Transport in Soil–Rice Systems: Novel Insights from Stable Isotope Fractionation and Gene Expression. Environ. Sci. Technol. **58**:12467–12476. https://doi.org/10.1021/acs.est.4c01851.

Zulfiqar, U., Jiang, W., Xiukang, W., Hussain, S., Ahmad, M., Maqsood, M.F., Ali, N., Ishfaq, M., Kaleem, M., Haider, F.U., et al. (2022). Cadmium Phytotoxicity, Tolerance, and Advanced Remediation Approaches in Agricultural Soils; A Comprehensive Review. Front. Plant Sci. **13**:773815. https://doi.org/10.3389/fpls.2022.773815.

Plant Communications, Volume 7

Supplemental information

A transposon insertion in the 5′ UTR of *OsPT1* reprograms its expression pattern and promotes cadmium accumulation in rice grains

Shasha Peng, Dan Wang, Jinling Liu, Su Jiang, Yuchen Xu, Yufei Deng, Xiaolong Zhou, Fangzhi Hu, Zhuo Liu, Ye Peng, Hejun Ao, Yinghui Xiao, Jiurong Wang, Junliang Zhao, Bin Liu, Keke Yi, Lianyang Bai, Guo-Liang Wang, and Houxiang Kang

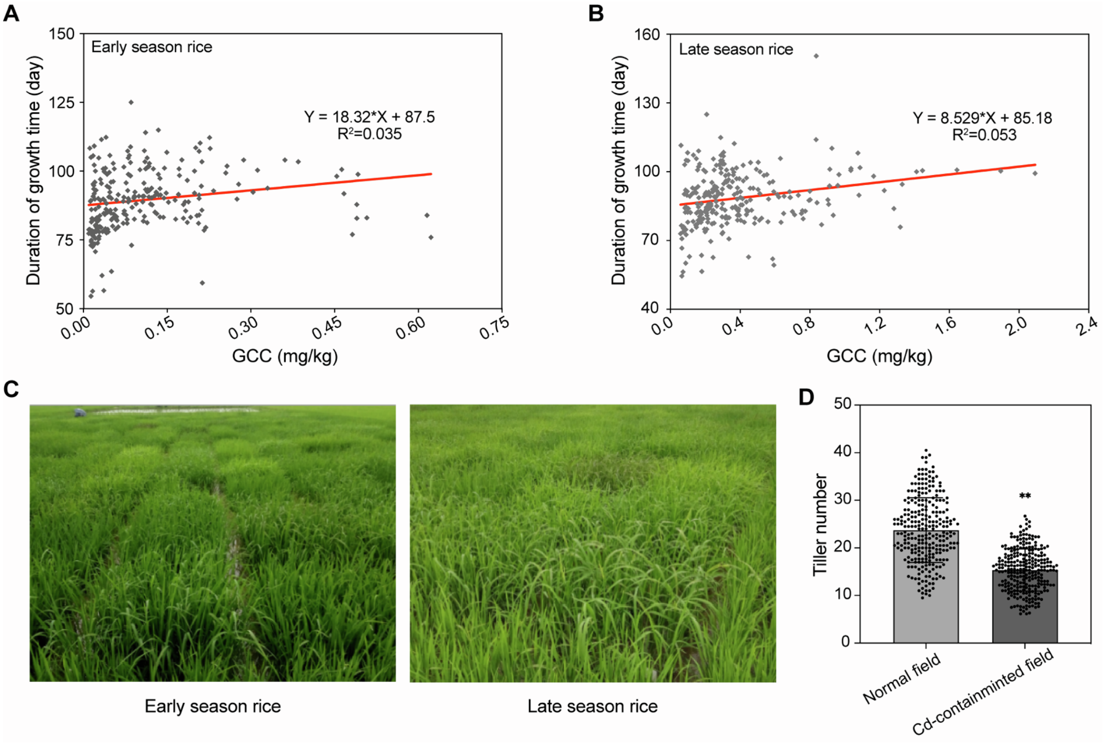

Figure S1. The correlation analysis between GCC and the growth period. **(A and B)** Scatter plots showing the relationship between GCC and growth duration for early-season rice **(A)** and late-season rice **(B)**. The fitted linear regression lines and corresponding equations are shown.

(**C**) Field photographs of RDP1 rice cultivars after transplanting: early-season rice (left), late-season rice (right).

(**D**) Tiller number of RDP1 cultivars grown in normal (left) and Cd-contaminated (right) fields. Each dot represents a single accession. Error bars represent SD; the asterisks denote significant differences according to Student's *t*-test (***P* <0.01).

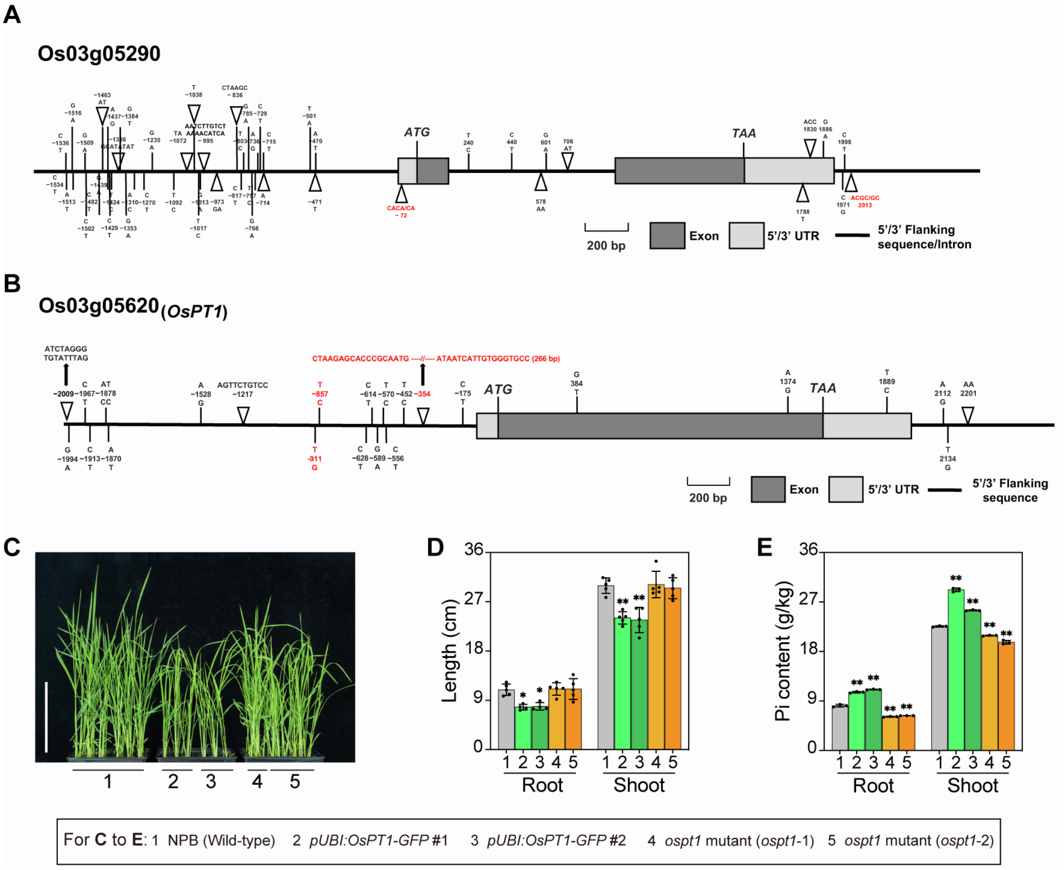

Figure S2. The polymorphisms were detected in two transporter genes in LAGCC4.

(A and B) Gene structure diagrams showing the polymorphisms identified by PCR and Sanger sequencing in Os03g05290 **(A)** and Os03g05620 (*OsPT1*) **(B)**. Black boxes indicate exons; gray boxes indicate 5′/3′ UTR; lines represent flanking sequences and intron.

(C) Representative phenotypes of 2-week-old wild-type, *ospt1* mutants, and *OsPT1* overexpression (*pUBI:OsPT1-GFP*) lines transferred to hydroponic growth conditions. Scale bar = 10 cm.

(D) *OsPT1* overexpression lines have shorter root and shoot lengths. Error bars represent SD; asterisks denote significant differences according to Student's *t*-test (**P* < 0.05, ***P* < 0.01).

(E) *OsPT1* overexpression lines show elevated phosphate (Pi) content in roots and shoots. In contrast, *ospt1* mutants have reduced Pi content in both tissues. Error bars represent SD; asterisks denote significant differences according to Student's t-test (**P* < 0.05, ***P* < 0.01).

A

Group	Accessions \ Position	ATG-1223	ATG-911	ATG-857	ATG-602	ATG-354	ATG+384	ATG+1374
High GCC accessions	*NSFTV_398*	8 bp +	T	T	C	266 bp +	G	A
	NSFTV_355	8 bp +	T	T	C	266 bp +	G	A
	NSFTV_353	8 bp +	T	T	C	266 bp +	G	A
	NSFTV_154	8 bp +	T	T	C	266 bp +	G	A
	NSFTV_156	8 bp +	T	T	C	266 bp +	G	A
	NSFTV_074	8 bp +	T	T	C	266 bp +	G	A
	NSFTV_072	8 bp +	T	T	C	266 bp +	G	A
	NSFTV_633	8 bp +	T	T	C	266 bp +	G	A
	NSFTV_076	8 bp +	T	T	C	266 bp +	G	A
	NSFTV_071	8 bp +	T	T	C	266 bp +	G	A
Low GCC accessions	*NSFTV_173*	-	G	C	C	-	T	G
	NSFTV_365	-	G	C	C	-	T	G
	NSFTV_064	-	G	C	C	-	T	G
	NSFTV_287	-	G	C	C	-	T	G
	NSFTV_177	-	G	C	C	-	T	G
	NSFTV_121	-	G	C	T	-	T	G
	NSFTV_629	-	G	C	C	-	T	G
	NSFTV_115	-	G	C	C	-	T	G
	NSFTV_224	-	G	C	C	-	T	G
	NSFTV_073	-	G	C	C	-	T	G
	NSFTV_033	-	G	C	C	-	T	G
	NSFTV_307	-	G	C	C	-	T	G
	NSFTV_265	-	G	C	C	-	T	G
	NSFTV_339	-	G	C	T	-	T	G
	NSFTV_091	-	G	C	C	-	T	G
	NSFTV_335	-	G	C	T	-	T	G

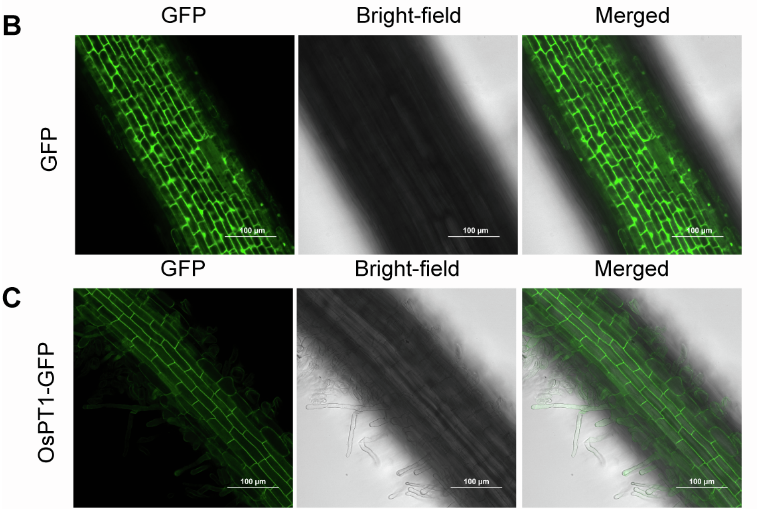

Figure S3. Haplotype variation and subcellular localization analysis of OsPT1.

(A) Haplotype analysis of *OsPT1* in 10 rice accessions with high GCC (in blue) and 16 rice accessions with low GCC (in green).

(B and C) Fluorescence microscopy showing subcellular localization of GFP **(B)** and OsPT1-GFP **(C)** in rice root tip cells.

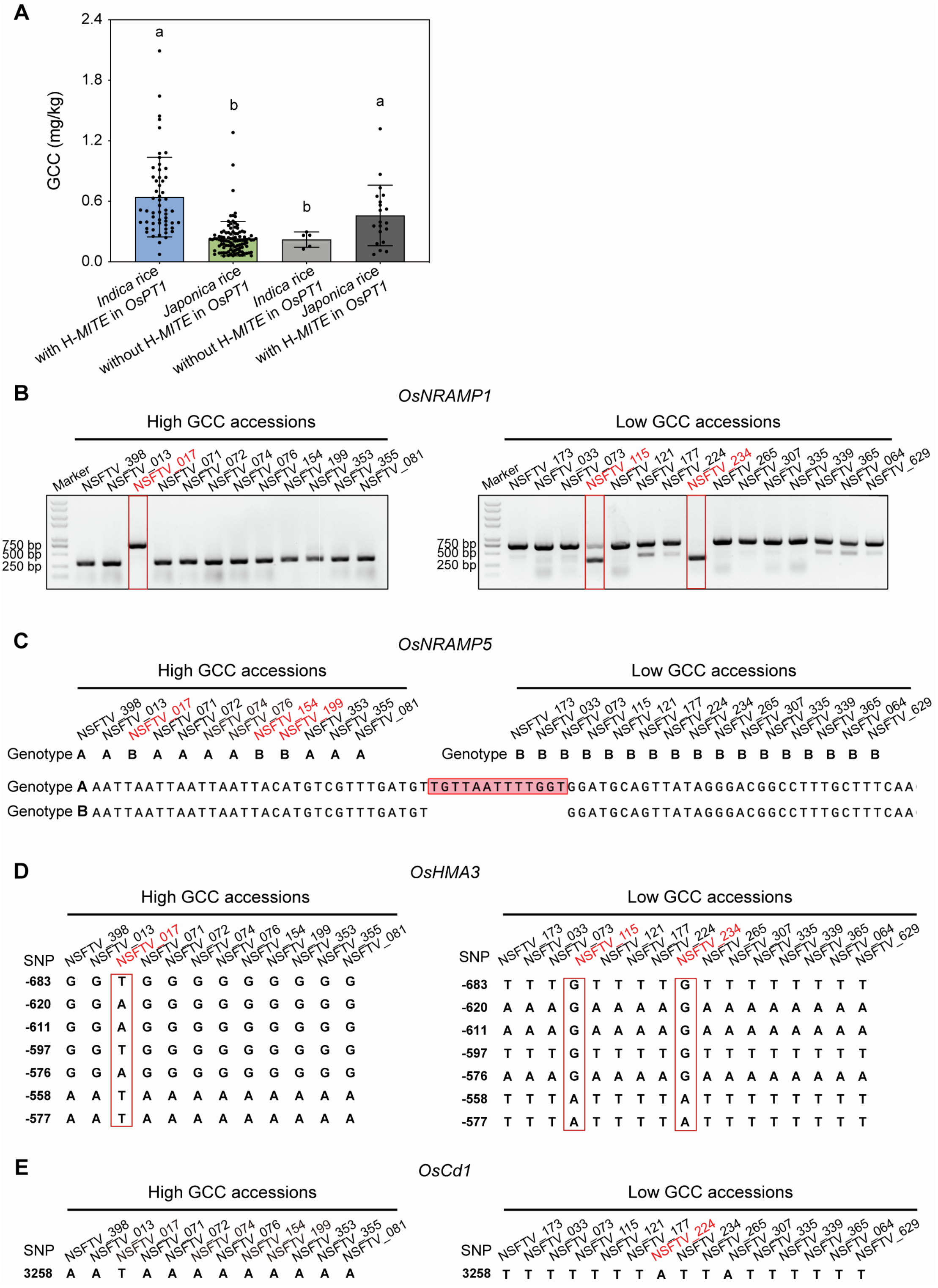

Figure S4. GCC of the RDP1 rice with or without H-*MITE* in *OsPT1* and genotype analysis of rice accessions for four key Cd-associated transporters.

(A) Histogram of average GCC for *Indica* rice with H-*MITE* in *OsPT1*, *Japonica* rice without H-*MITE* in *OsPT1, Indica* rice without H-*MITE* in *OsPT1,* and *Japonica* rice with H-*MITE* in *OsPT1*. Error bars represent SD; the "a" and "b" denote significant differences, as determined by one-way ANOVA with Tukey's HSD test.

(B) Genotypes of *OsNRAMP1* in representative accessions with high (left) and low (right) GCC, shown by gel electrophoresis.
(C) Genotypes of *OsNRAMP5* in high GCC accessions (left) and low GCC accessions (right). A and B represent the different genotypes, respectively.
(D) Genotypes of *OsHMA3* in high GCC accessions (left) and low GCC accessions (right).
(E) Genotypes of *OsCd1* in high GCC accessions (left) and low GCC accessions (right).

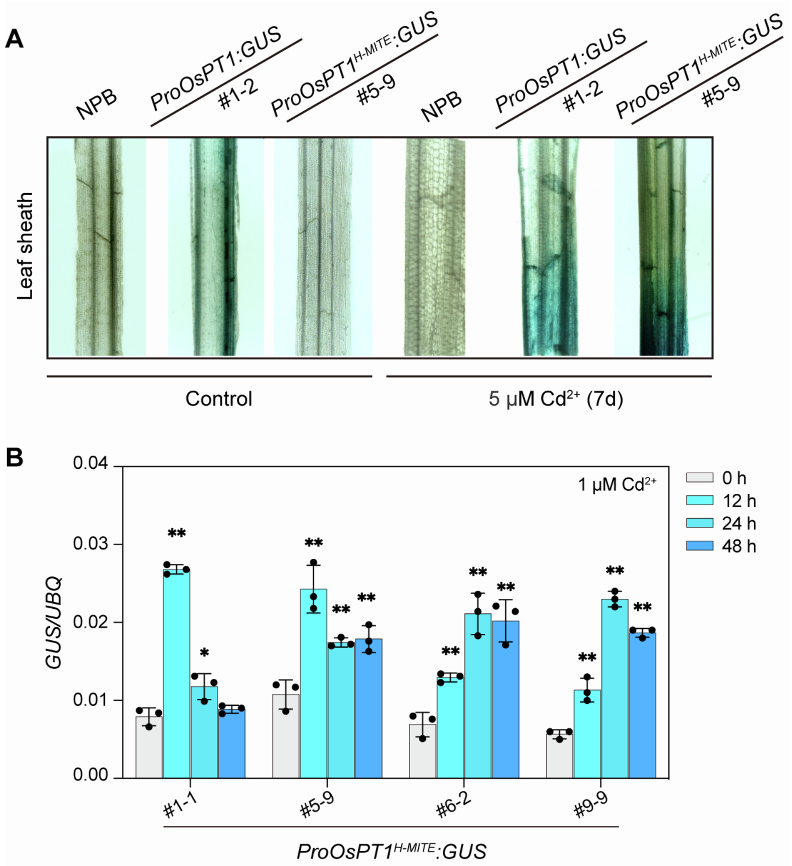

Figure S5. Cd treatment induces *GUS* transcription in stable transgenic rice plants of *ProOsPT1$^{H\text{-}MITE}$*:*GUS* but not *ProOsPT1*:*GUS*.

(A) GUS staining of leaf sheaths in wild-type (NPB), *ProOsPT1*:*GUS* and *ProOsPT1$^{H\text{-}MITE}$*:*GUS* transgenic seedlings; images on the left are from the control (no Cd treatment), images on the right are from seedlings exposed to Cd for 7 days.

(B) qRT-PCR analysis of GUS expression in four independent *ProOsPT1$^{H\text{-}MITE}$* T_2 lines (#1-1, #5-9, #6-2 and #9-9) under 1 μM Cd^{2+} stress condition. Error bars indicate SD; asterisks denote significant differences compared with 0 h (*P < 0.05, **P < 0.01, *t*-test). Different shades represent different Cd exposure durations.

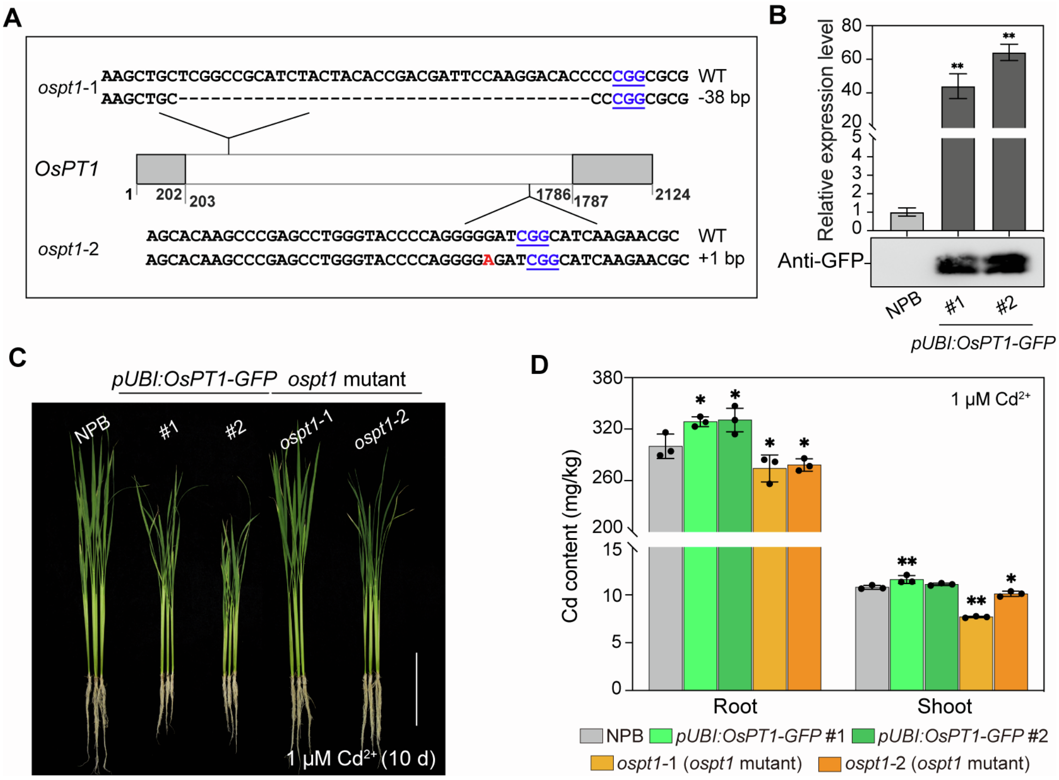

Figure S6. Overexpression of *OsPT1* causes high Cd accumulation in rice seedlings.

(A) Targeted mutagenesis of *OsPT1* using CRISPR-Cas9 mediated genome editing. Two independent gene editing sites were designed (sgRNA sequences are given in the figure). Two homozygous mutants (*ospt1-1* and *ospt1-2*) were obtained, harboring a 38-bp deletion (*ospt1-1*) or a 1-bp insertion (*ospt1-2*).
(B) *OsPT1* transcript levels and OsPT1 protein abundance in transgenic plants *OsPT1* overexpressing (*pUBI:OsPT1-GFP*#1 and *pUBI:OsPT1-GFP*#2). Protein abundance was determined by immunoblot with anti-GFP antibody.
(C and D) Phenotypes of wild type, *ospt1* mutant, and *OsPT1*-overexpressing (*pUBI:OsPT1-GFP*) lines, 2-week-old rice seedlings transferred to hydroponic growth conditions with 1 μM Cd^{2+} for 10 days **(C)**. Cd content in roots and shoots after treatment with 1 μM Cd^{2+} for 10 days in *OsPT1*-overexpressing and *ospt1* mutant plants **(D)**. Error bars represent SD; asterisks denote significant differences according to Student's *t*-test (**P* < 0.05, ***P* < 0.01).

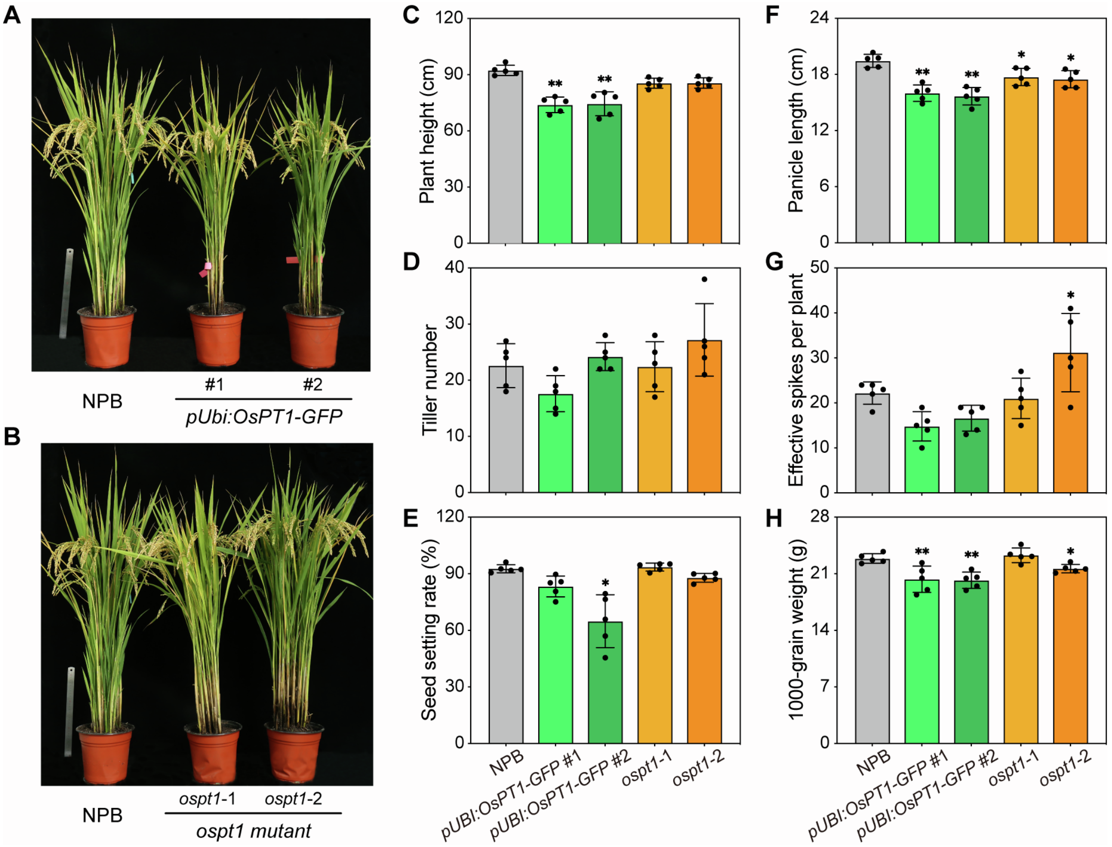

Figure S7. The agronomic traits of *OsPT1* overexpression and *ospt1* mutant plants.

(A and B) Phenotypes of wild type, *OsPT1*-overexpression **(A)**, and *ospt1* mutant **(B)** plants grown in the soil.

(C to H) The agronomic traits of wild type, *OsPT1*-overexpression, and *ospt1* mutant plants. Plant height **(C)**, tiller number **(D)**, seed setting **(E)**, panicle length **(F)**, effective spikes per plant **(G)**, and 1000-grain weight **(H)**. Error bars represent SD; asterisks denote significant differences according to Student's *t*-test (*$P < 0.05$, **$P < 0.01$).

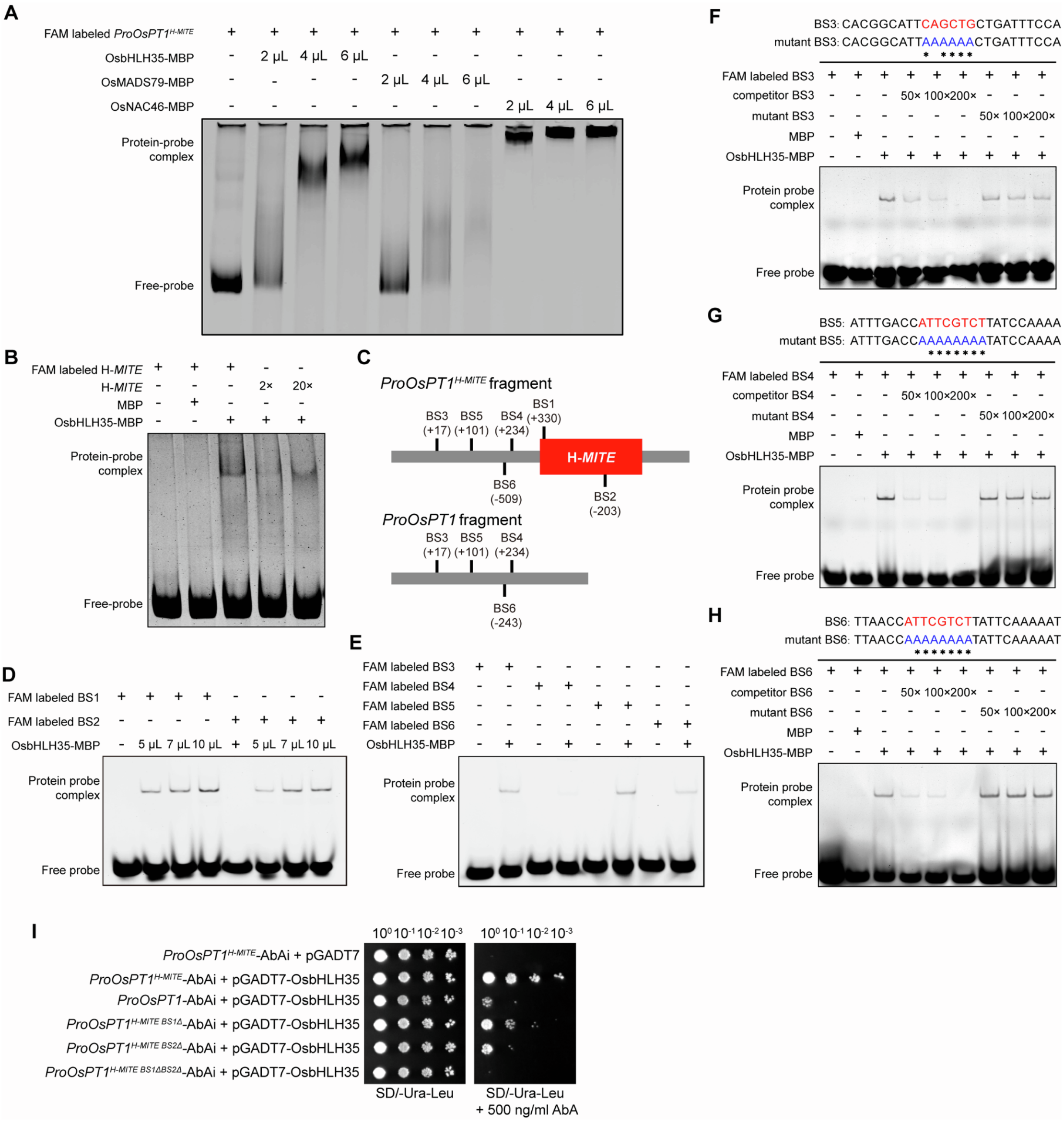

Figure S8. OsbHLH35 binds directly to *ProOsPT1$^{H\text{-}MITE}$*.

(A) EMSAs showing binding of OsbHLH35-MBP and OsNAC46-MBP proteins to FAM-labeled *ProOsPT1$^{H\text{-}MITE}$* probe. MBP serves as a negative control.

(B) EMSAs demonstrating direct binding of OsbHLH35 to the H-*MITE* sequence. Competitor probes were added at 2- and 20-fold excess.

(C) Schematic representation of predicted OsbHLH35 binding sites (BS1–BS6) on *ProOsPT1$^{H\text{-}MITE}$* and in *ProOsPT1.*

(D) EMSAs showing that OsbHLH35 can bind to BS1 and BS2 (located in the H-*MITE*).

(E) EMSAs showing that OsbHLH35 can bind to BS3, BS5, and BS6.

(F to H) EMSAs to confirm the binding of OsbHLH35 to the three binding sites in the *OsPT1$^{H\text{-}MITE}$* promoter. The competitor probe and mutant probe were added at 50-, 100-, and 200-fold molar excess of labeled probes, respectively.

(I) Yeast one-hybrid showing direct binding of OsbHLH35 to the *OsPT1$^{H\text{-}MITE}$* promoter.

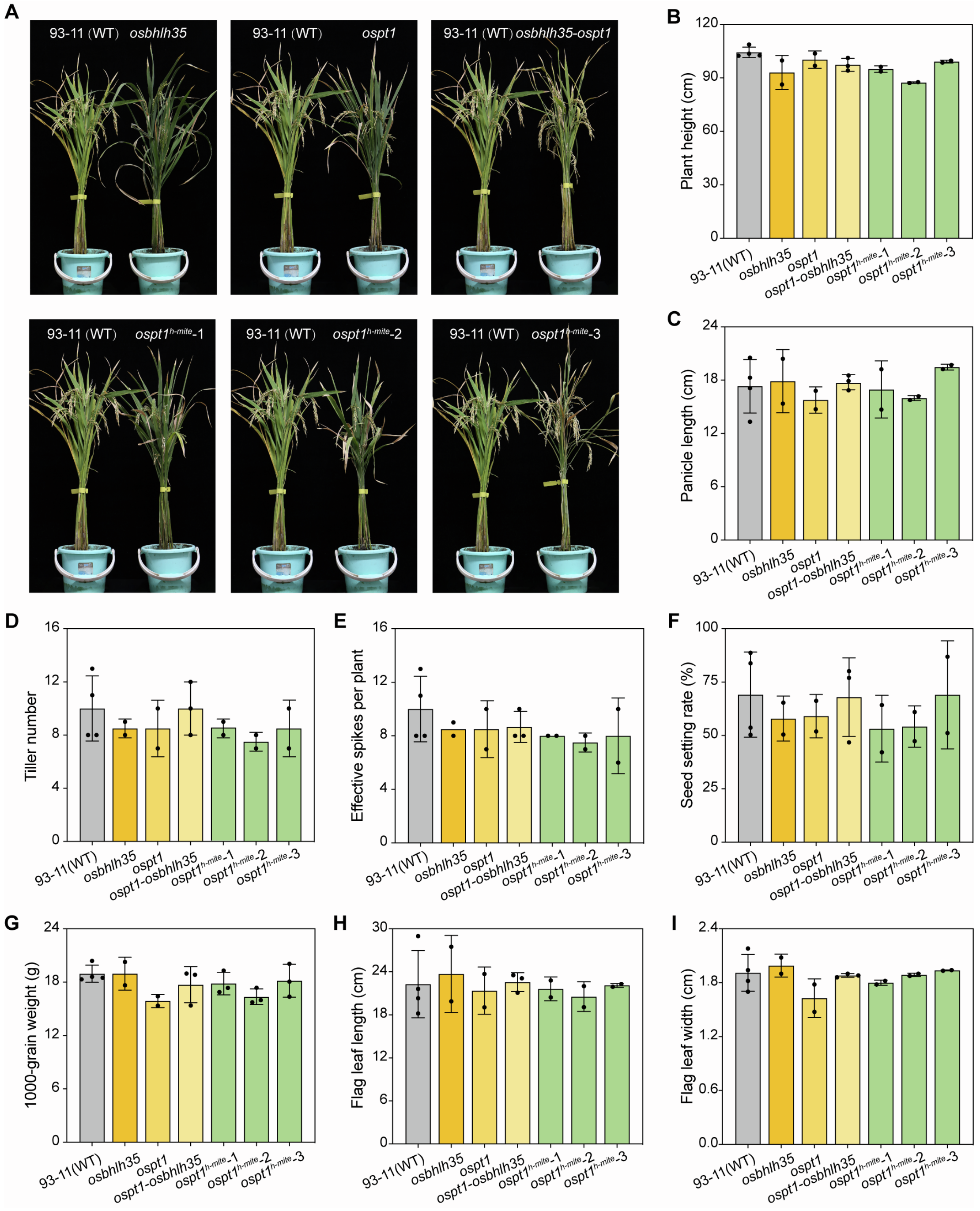

Figure S9. The agronomic traits were evaluated in *ospt1*, *osbhlh35*, and *ospt1$^{h\text{-}mite}$* knockout mutant plants.

(A) Phenotypes of wild type 93-11, *osbhlh35*, *ospt1*, *osbhlh35-ospt1,* and *ospt1$^{h\text{-}mite}$* mutant plants.

(B to I) The agronomic traits of wild type, *ospt1, osbhlh35, ospt1-osbhlh35,* and *ospt1$^{h\text{-}mite}$* mutant plants. Plant height **(B)**, panicle length **(C)**, tiller number **(D)**, effective spikes per plant **(E)**, seed setting rate **(F)**, 1000-grain weight **(G)**, flag leaf length **(H)**, and flag leaf width **(I)**. Error bars represent SD; asterisks denote significant differences according to Student's *t*-test (*$P < 0.05$, **$P < 0.01$).

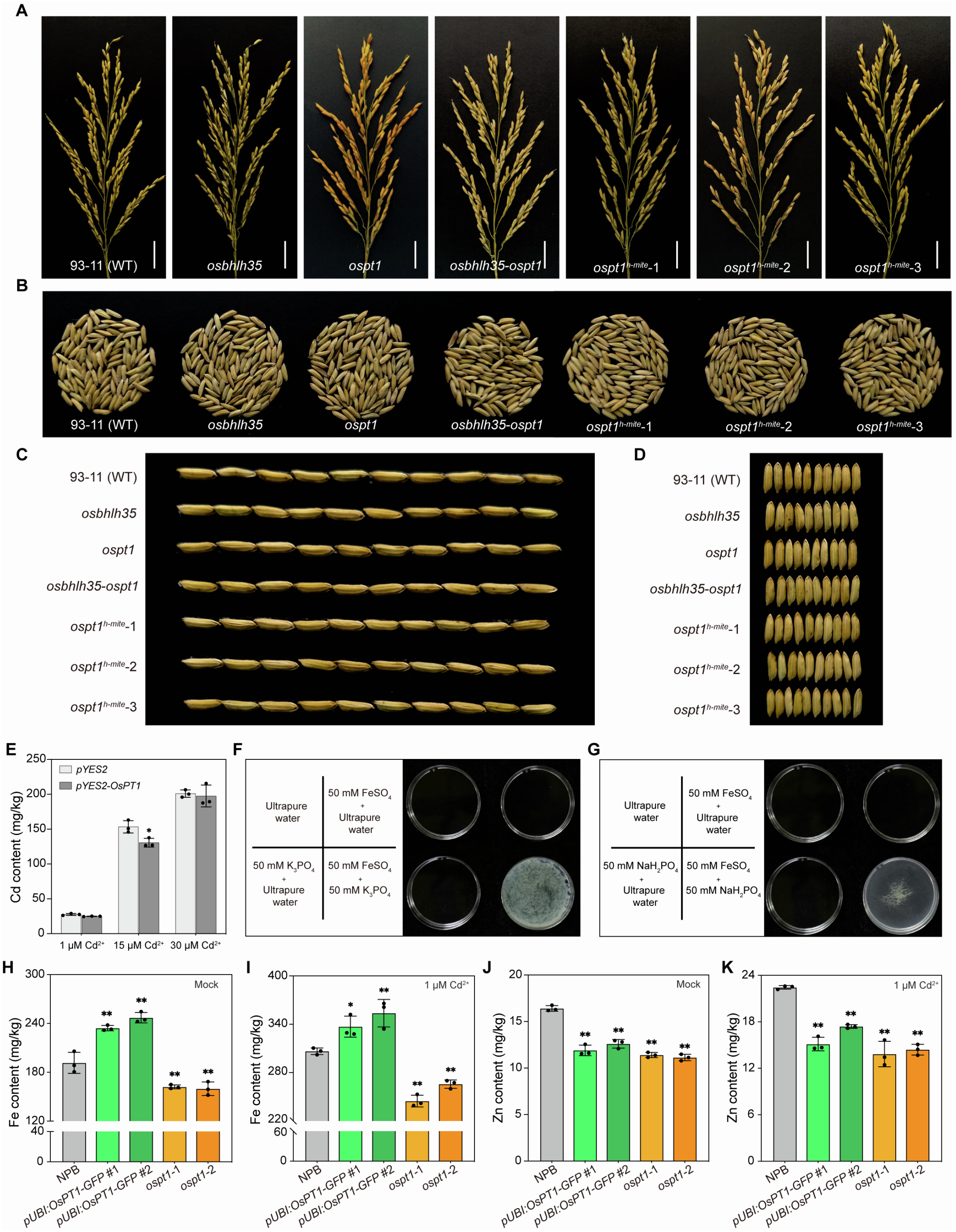

Figure S10. Phenotypes of *osbhlh35*, *ospt1*, and *in vitro* assay to test the interaction between phosphate and different ions.

(A to D) Representative seed phenotypes of wild-type 93-11, *osbhlh35*, *ospt1*, *osbhlh35-ospt1,* and *ospt1*$^{h\text{-}mite}$ mutants' plants. Scale bars = 5 cm.

(E) Cd content in wild type yeast strain *INVSc1* (empty vector: *pYES2*) and *INVSc1* expressing *OsPT1* (*pYES2-OsPT1*) after being treated with Cd^{2+} for 24 hours. Error bars represent SD; asterisks denote significant differences according to Student's *t*-test (*$P < 0.05$, **$P < 0.01$).

(F and G) In *vitro* assay to test the interaction between phosphate and Fe^{2+}.

Both $(PO_4)^{3-}$ (F) and $(H_2PO_4)^-$ (G) can strongly bind to Fe^{2+} and quickly react to form the precipitate within 5 minutes.

(H and I) Fe content of the rice shoots under normal **(H)** and 1 μM Cd^{2+} stress **(I)** conditions in *OsPT1* overexpression and *ospt1* mutant plants. The error bars represent SD; the asterisks denote significant differences according to Student's *t*-test (*$P < 0.05$, **$P < 0.01$).

(J and K) Zn contents of the rice shoots under normal **(J)** and 1 μM Cd^{2+} stress **(K)** conditions in *OsPT1* overexpression and *ospt1* mutant plants. Error bars represent SD; asterisks denote significant differences according to Student's *t*-test (*$P < 0.05$, **$P < 0.01$).
